# Postoperative outcomes after arthroscopic repair of isolated subscapularis tears: a systematic review and meta-analysis

**DOI:** 10.1186/s13018-026-07088-5

**Published:** 2026-07-17

**Authors:** Ana Belén Fernández-Cortiñas, Teresa Seoane-Pillado, Yaiza Lopiz, Miguel Hermida, J. R. Caeiro-Rey, Fernando Marco

**Affiliations:** 1 Traumatology and Orthopaedic Surgery Department, Santiago de Compostela University Hospital, Santiago de Compostela, Spain; 2 Traumatology and Orthopaedic Surgery Department, Recoletas Cosaga Hospital, Ourense, Spain; 3 Preventive Medicine and Public Health Unit, Department of Health Sciences, University of A Coruña-INIBIC, A Coruña, Spain; 4 Shoulder and Elbow Unit, Traumatology and Orthopaedic Surgery Department, Clínico San Carlos Hospital, Madrid, Spain; 5 Surgery Department, Complutense University, Madrid, Spain

**Keywords:** Subscapularis tear, Arthroscopic repair, Rotator cuff, Shoulder, Meta-analysis

## Abstract

**Purpose:**

To systematically synthesize pre- and postoperative outcomes of arthroscopic repair for isolated subscapularis (SSC) tears across functional scores, pain, strength, range of motion (ROM), and clinical tests.

**Study design:**

Systematic review and meta-analysis.

**Methods:**

A systematic literature search was conducted across PubMed, Cochrane Library, and SCOPUS for studies reporting pre- and postoperative outcomes after arthroscopic repair of isolated SSC tears in adults with a minimum 12-month follow-up. Data on functional outcome scores, pain measures, and subscapularis-specific clinical tests were assessed. Functional outcomes were assessed using validated shoulder scoring systems, including the Constant–Murley Score (CMS), University of California, Los Angeles Shoulder Score (UCLA), American Shoulder and Elbow Surgeons Score (ASES), Simple Shoulder Test (SST), and Subjective Shoulder Value (SSV). These scores generally evaluate a combination of pain, shoulder function, strength, and activities of daily living. Pain was assessed using the Visual Analogue Scale (VAS). Subscapularis-specific function was evaluated using clinical tests such as the lift-off, belly-press, and bear-hug tests. The outcomes were presented as a mean difference between the preoperative and postoperative values using random or fixed effects. Results are reported as mean difference (MD) or risk ratio (RR) with 95% confidence interval (CI) and exact P values. Certainty of evidence was evaluated using the GRADE approach. The evidence base consisted predominantly of non-randomized studies at serious risk of bias, with outcomes rated as low or very low certainty.

**Results:**

Seventeen non-randomized studies (Level II–IV evidence) comprising 537 patients were included. In the absence of a control group, mean postoperative functional outcome scores were higher than preoperative values across studies, although individual patient outcomes varied. All included studies were judged to be at serious risk of bias using the ROBINS-I tool, and GRADE certainty of evidence was low to very low for all outcomes. Improvements were reported in multiple domains including shoulder function, strength, ROM, pain, and ability to perform daily activities. The mean differences ranged from 16.9 to 45.8 points for the CMS (pooled MD = 31.4, 95% CI [27.0–35.9]), 14.5 to 16.0 points for the UCLA (pooled MD = 15.4, 95% CI [14.8–15.9]), 33.0 to 58.3 points for the ASES (pooled MD = 48.1, 95% CI [46.8–49.9]), and 4.3 to 5.1 points for the SST (pooled MD = 4.9, 95% CI [4.4–5.5]). Consistent improvement was also observed in the SSV (pooled MD = 48.2, 95% CI [46.7–49.8]). In addition, pain assessed using the Visual Analogue Scale showed a significant postoperative reduction (MD =  − 5.0, 95% CI [− 5.2 to − 4.8]). Subscapularis-specific clinical tests showed a significant decrease in the rate of positive lift-off (RR = 0.19, 95%CI (0.14–0.27)), belly-press (RR = 0.36, 95% CI (0.29–0.46)), and bear-hug tests (RR = 0.46, 95% CI (0.35–0.61)). Furthermore, ROM, which was primarily assessed clinically through measures of internal rotation was evaluated using either the Constant–Murley internal rotation subscore or spinal level assessment scales, depending on the study, improved significantly postoperatively. Subscapularis strength, evaluated clinically using lift-off and/or belly-press strength testing, most commonly graded according to the Medical Research Council (MRC) scale ranging from 0 to 5, also demonstrated significant postoperative improvement. Although overall postoperative strength improved significantly at the study level, some patients continued to demonstrate residual weakness or persistent positive subscapularis-specific clinical tests following repair. A major confounder was the frequent co-performance of long head of the biceps tenotomy or tenodesis (reported in 55.6–100% of cases across studies), which limits the ability to attribute postoperative improvements to subscapularis repair alone.

**Conclusion:**

Arthroscopic repair of isolated SSC tears was associated with postoperative improvements in pain, functional scores, range of motion, and subscapularis-specific clinical tests in this predominantly observational evidence base. However, these findings should be interpreted with caution, as the certainty of evidence was low to very low for all outcomes (GRADE), all included studies were at serious risk of bias (ROBINS-I), and the frequent co-performance of long head of the biceps procedures (tenotomy or tenodesis) represents a major confounder that limits attribution of improvements to subscapularis repair alone. The durability of these outcomes and the extent to which muscle strength is restored remain uncertain, particularly in the long term, and may be limited by pre-existing muscle degeneration.

**Supplementary Information:**

The online version contains supplementary material available at https://doi.org/10.1186/s13018-026-07088-5.

## Introduction

The subscapularis (SSC) is the most powerful rotator cuff (RC) muscle, contributing to 53% of the cuff moment [1, 2]. SSC involvement has been reported in approximately 18% of rotator cuff pathology, whereas isolated SSC tears represent only 4–6% of all RC tears [1, 2].

Most SSC tears are degenerative and occur in association with massive RC lesions. In contrast, isolated SSC tears are more commonly associated with acute traumatic injury in younger patients, particularly following forced external rotation or extension with the arm abducted. Clinically, isolated SSC tears commonly present with anterior shoulder pain [3, 4]. Although the subscapularis tendon was historically referred to as the ‘forgotten tendon,’ advances in imaging modalities and the increasing use of arthroscopy have improved the detection and surgical management of SSC tears. These advances have also enhanced the characterization of different tear patterns, including isolated SSC tears, anterosuperior cuff tears, and SSC tears associated with large rotator cuff lesions [5–8].

The current standard management of isolated SSC tears follows a gradual approach based on tear severity and patient-related factors [9]. Mild fraying involving the upper third of the tendon can be treated conservatively with physical therapy, activity modification, nonsteroidal anti-inflammatory drugs (NSAIDs), and/or corticosteroid injections, whereas more substantial full-thickness tears typically require surgical intervention due to SSC tendon retraction and fatty infiltration [9, 10]. Several studies have reported comparable outcomes between operative and conservative management for isolated SSC tears, particularly in patients with advanced fatty infiltration [11]. However, when surgical intervention is indicated, arthroscopic repair has emerged as the preferred operative technique, demonstrating outcomes comparable to open repair while offering several minimally invasive advantages, including improved intra-articular visualization, preservation of the deltoid and surrounding soft tissues, reduced soft-tissue disruption, lower postoperative morbidity, and potentially lower infection risk [5–8, 10].

Despite their low prevalence, isolated SSC tears are clinically important because the subscapularis is the largest muscle of the rotator cuff and plays a critical role in shoulder stability, internal rotation, force coupling, and anterior glenohumeral joint stabilization. Isolated SSC tears present unique diagnostic and therapeutic challenges owing to their frequently delayed diagnosis, variable clinical presentation, association with long head of the biceps tendon pathology, and potential progression to fatty infiltration and muscle degeneration. Moreover, postoperative outcomes following isolated SSC repair may differ from those of posterosuperior rotator cuff tears because of their distinct anatomical and biomechanical characteristics. Therefore, evaluating isolated SSC tears separately minimizes confounding from concomitant rotator cuff pathology and allows a more accurate assessment of the clinical and functional outcomes specifically attributable to SSC repair.

Previous meta-analyses have evaluated isolated SSC injuries; however, these have primarily focused on diagnostic approaches or risk factors rather than treatment outcomes [12, 13]. While some systematic reviews have addressed the results of arthroscopic repair for isolated SSC tears, [14–16] at the time of the final literature search, no quantitative meta-analyses were evaluating the outcomes of arthroscopic repair in isolated SSC tears in the current literature search.

This meta-analysis aimed to synthesize the pre- and postoperative outcomes of arthroscopic repair for isolated SSC tears across the Constant-Murley Score (CMS), University of California Los Angeles Shoulder Score (UCLA), American Shoulder and Elbow Surgeons Score (ASES), Simple Shoulder Test (SST), and Subjective Shoulder Value (SSV), pain (visual analog scale [VAS]), and subscapularis-specific clinical tests (lift-off, belly-press, and bear-hug tests), acknowledging the non-comparative, observational nature of the available evidence.

## Methods

This systematic review and meta-analysis was prospectively registered on PROSPERO (CRD42025643733) and follows the recommendations of the PRISMA (Preferred Reporting Items for Systematic Reviews and Meta-Analyses) guidelines [17], in accordance with the Cochrane Handbook for Systematic Reviews of Interventions [18].

### Literature search

A comprehensive literature search was conducted in the PubMed, Cochrane Library, and SCOPUS databases for articles published between 2000 and 2025. This broad time window was selected to maximize retrieval of eligible studies because isolated SSC tears are relatively rare. The search strategy was based on the application of Boolean logic operators to the keywords presented in Additional File 1. Additionally, the reference lists of all included articles were manually screened to identify any potentially relevant studies that may have been missed during the database search.

Following the search, all retrieved records were imported into EndNote software (Clarivate Analytics, PA, USA) for duplicate removal. The remaining references were then uploaded to the Rayyan website [19], where two reviewers independently conducted a two-step screening process. This involved an initial screening of titles and abstracts, followed by a full-text assessment of the articles deemed potentially eligible. Any disagreements during the screening process were resolved through discussion.

### Eligibility criteria

We included prospective or retrospective cohort and case series studies that focused on adult patients (≥ 18 years) who underwent arthroscopic repair for isolated SSC tendon tears. Studies were included if they provided extractable data for adult patients and reported a preoperative–postoperative comparison with a minimum follow-up period of 12 months.

The included studies reported arthroscopic repair of isolated subscapularis tendon tears with the primary aim of restoring the tendon’s anatomical footprint. Surgical techniques varied across studies, including differences in repair configuration (single-row versus double-row constructs), number and type of suture anchors, and management of concomitant intra-articular pathology, particularly long head of the biceps tendon lesions treated with tenotomy or tenodesis. Although anatomical restoration was the shared operative goal, the surgical approaches were not fully standardized across the included studies. Postoperative rehabilitation protocols also varied but generally consisted of an initial period of immobilization, followed by staged progression from passive to active-assisted range of motion, with strengthening introduced in later phases. The implications of this variability are addressed in the limitations section.

Eligible studies reported at least one functional outcome score, pain measurement, or subscapularis-specific clinical test. Functional outcomes were assessed using validated shoulder scoring systems, including the CMS, ASES, UCLA Shoulder, SST, and SSV scores. These instruments evaluate multiple clinical domains, including pain, shoulder function, strength, range of motion, and activities of daily living. The CMS, ASES, and UCLA scores are composite measures combining patient-reported and clinician-assessed components, with higher scores indicating better shoulder function. The SST and SSV are patient-reported measures of perceived shoulder performance, with higher values reflecting better outcomes. Pain intensity was assessed using the VAS, with lower scores indicating reduced pain severity.

Subscapularis strength was primarily evaluated using clinical examination-based tests, including the lift-off, belly-press, and bear-hug tests. These were most graded using the Medical Research Council (MRC) scale (0–5) or reported as categorical outcomes (positive/negative or graded improvements). In most studies, strength outcomes were based on clinical testing rather than objective dynamometric measurements; therefore, reported strength improvements reflect clinical performance rather than instrumented quantitative force assessment.

ROM and internal rotation outcomes were assessed using heterogeneous methods in the included studies. Most studies measured shoulder ROM using goniometric techniques and reported angular values for flexion, abduction, and external rotation. Internal rotation was most commonly assessed using either the internal rotation subscore of the Constant–Murley Score or spinal level–based grading (hand-behind-back function). In a minority of studies, ROM outcomes were embedded within composite functional scores rather than reported as stand-alone continuous variables.

Owing to this variability, only studies reporting extractable continuous or comparable quantitative data were included in the pooled analyses. Ordinal or non-standardized ROM measures were descriptively summarized. Where applicable, changes in functional outcomes were interpreted in relation to published minimal clinically important difference (MCID) thresholds to enhance clinical interpretability.

Studies were excluded if they involved pediatric populations, had a follow-up of less than 12 months, or included patients with prior shoulder surgeries or concomitant shoulder injuries. Studies in which additional major concomitant shoulder procedures or mixed surgical populations prevented isolation of outcomes specific to isolated SSC repair were excluded. However, studies in which common adjunct procedures performed as part of SSC repair (particularly long head of biceps tenotomy or tenodesis) were included, as these are frequently considered part of the standard surgical management of SSC pathology rather than separate interventions. We also excluded case reports, reviews, letters, conference abstracts, and studies with incomplete datasets. Only studies published in English were included. Non-English publications were excluded due to limitations in translation and data extraction resources.

### Data collection process

Data were extracted independently by two reviewers using a standardized collection sheet. For each included study, the following information was collected: first author, publication year, study design, level of evidence, study period, follow-up duration, sample size, patient age and sex, and all relevant clinical outcomes, including CMS, UCLA, ASES, SST, SSV, VAS, lift-off test, belly-press test, bear-hug test, active ROM in internal rotation, and internal rotation strength.

### Quality of evidence assessment

Evidence quality for all outcomes was rated according to the Grading of Recommendations Assessment, Development and Evaluation (GRADE) approach [20]. The certainty of evidence in the GRADE approach is assessed across five aspects: risk of bias, inconsistency, indirectness, imprecision, and publication bias. For each area, a systematic assessment was made as “unserious,” “serious,” or “very serious.” The overall level of evidence was ranked as high (⊕ ⊕  ⊕ ⊕), moderate (⊕ ⊕  ⊕ ○), low (⊕ ⊕ ○○) or very low (⊕ ○○○).

### Assessment of risk of bias in included Studies

The risk of bias in the included non-randomized studies was assessed using the Risk Of Bias In Non-randomized Studies of Interventions (ROBINS-I) tool. This tool evaluates bias across seven domains: (1) bias due to confounding, (2) bias in selection of participants into the study, (3) bias in classification of interventions, (4) bias due to deviations from intended interventions, (5) bias due to missing data, (6) bias in measurement of outcomes, and (7) bias in selection of the reported result. Each domain was judged as ‘low risk’, ‘moderate risk’, ‘serious risk’, ‘critical risk’, or ‘no information’. The overall risk of bias for each study was determined based on the most severe judgement across all domains. Two reviewers independently assessed the risk of bias, and disagreements were resolved through discussion or consultation with a third reviewer. The results are presented in a traffic light plot showing domain-level judgements for each study and a summary bar chart showing the distribution of judgements across studies.

### Statistical analysis

All meta-analyses were performed using R (v4.1.2) with the “meta”, “metafor”, “forestplot”, and ‘ggplot2’ packages. For continuous outcomes, the Mean Difference (MD) and standard deviations (SD) with 95% confidence intervals (CI) were calculated. For dichotomous outcomes, Risk Ratios (RR) with 95% CIs were calculated using the Mantel–Haenszel method. All statistical tests were two-tailed with α = 0.05. A *p*-value < 0.05 was considered statistically significant.

Heterogeneity between studies was assessed using the I2 statistic. For outcomes with low heterogeneity (I2 < 25%), a fixed-effects model was used. For outcomes with moderate (25–50%) or high (> 50%), a random-effects model using the DerSimonian and Laird method was applied. A pre-post correlation coefficient of 0.5 was assumed for the calculation of change-from-baseline standard deviations in paired pre- and post-analyses. This value reflects a moderate assumed correlation and is the conventional default applied in meta-analytic methods for continuous pre-post outcomes, as recommended by the Cochrane Handbook for Systematic Reviews of Interventions. The robustness of the pooled estimates to alternative assumed correlations (e.g., 0.3 or 0.7) was not formally tested in the present analysis.

### Publication bias

Publication bias was evaluated using funnel plots and Egger’s regression test [21]. Funnel plots were enhanced with contour-based significance testing to help in visual interpretation. An Egger’s test* p*-value < 0.05 was considered indicative of potential publication bias, and results for such outcomes were interpreted with appropriate caution.

### Subgroup analysis

Subgroup analyses were conducted to evaluate the effect of follow-up duration on outcomes. The CMS outcome was analyzed in three subgroups (< 24 months, 24–48 months, and > 48 months).

### Robustness of evidence

To test the robustness of the results, sensitivity analyses were performed using a leave-one-out approach, where each study was sequentially removed from the meta-analysis. An additional sensitivity analysis was conducted by excluding studies with low methodological quality.

## Results

### Literature search

**Fig. 1:**
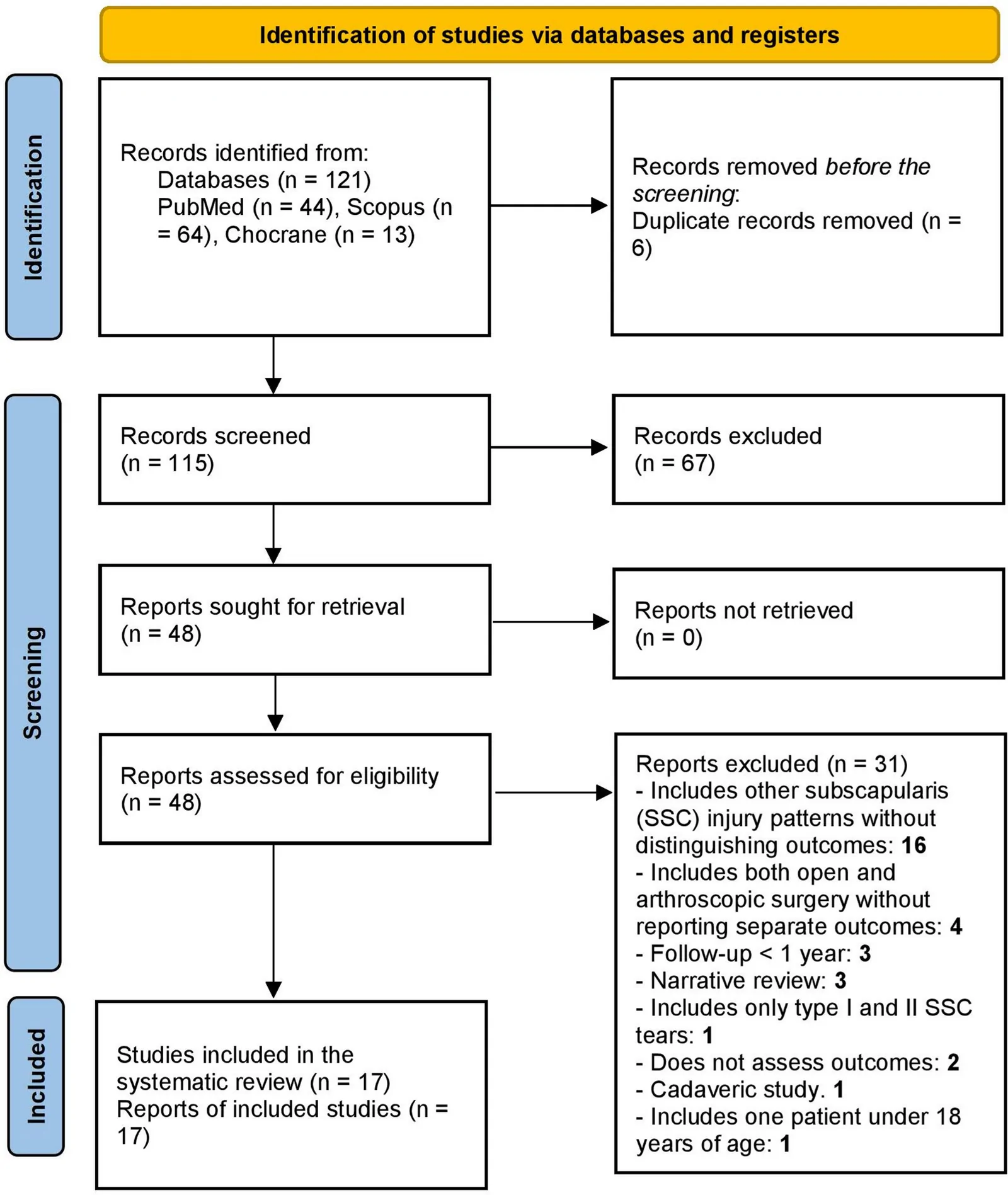
The PRISMA flow diagram of study selection process

The initial database search yielded 121 records. After the removal of 6 duplicates, 115 articles were screened based on their titles and abstracts. From this, 67 records were excluded for the following reasons: studies including patients with rotator cuff pathology other than isolated SSC tears (n = 17), review articles (n = 21), case reports (n = 14), small case series (n = 9), and non-English publications (n = 6). Finally, 48 articles were screened for full-text review. No articles were irretrievable. During the full-text eligibility assessment, a further 31 articles were excluded for various reasons which are detailed in the PRISMA flow diagram (Fig. 1). Finally, 17 studies met all inclusion criteria and were included in our systematic review and analysis. The complete study selection process is detailed in the PRISMA flow diagram (Fig. 1).

### Characteristics of the included studies

The 17 included studies were published between 2003 and 2024 and collectively enrolled a total of 537 patients undergoing isolated SSC repair [5, 11, 21–35]. The studies originated from various countries across Europe, Asia, and the Americas, including Germany, USA, Italy, South Korea, Switzerland, France, Japan, Brazil, and Turkey. According to analysis using the Oxford Centre for Evidence-Based Medicine criteria, most studies represented Level IV evidence (n = 11, 64.7%), with five studies providing Level III evidence (29.4%) and one study providing Level II evidence (5.9%). The mean follow-up duration varied considerably across the studies, ranging from a minimum of 12 months to a mean of 107 months. Patients were mostly male (70.1%), with a mean age at the time of surgery across the included studies 52.9 ± 6.6 years, ranging from 42.0 to 59.0 years. All studies assessed tears using an established classification system, most commonly the Lafosse classification for tear size. A consistent finding across the literature was a high incidence of concomitant long head of the biceps tendon pathology, which was frequently addressed with either tenotomy or tenodesis at the time of the SSC repair. The baseline characteristics of all included studies are summarized in Table 1.

**Table 1:** Baseline characteristics of included studies

References	Country	Study design	Level of evidence	Study interval	Follow-up mean (months)	Patients—shoulders (n)	Age at surgery (mean years ± SD)	Male/Female	COI (yes/no)	Funding (yes/no)
Bartl [21]	Germany	PC	III	2012 to 2020	52	18	51.0	14/4	No	No
Bartl [5]	Germany	CS	IV	2003 to 2006	27	21	43.7	16/5	Yes	Yes
Bennett [22]	USA	PC	II	1995 to 1999	24–48	8	56.8	5/3	No	No
Galasso [23]	Italy	CS	IV	2005 to 2020	107	45	55.0	39/6	No	No
Godinho [24]	Brazil	CS	IV	2003 to 2009	34	18	59.0	13/5	No	No
Grueninger [25]	Switzerland	CS	IV	2008 to 2010	12	11	45.0	9/2	No	No
Hasler [26]	Switzerland	CS	IV	2003 to 2012	103	36	57.7	21/15	No	No
Heikenfeld [27]	Germany	CS	IV	2006 to 2008	24	20	42.0	18/2	No	No
Kamijo [28]	Japan	RC	III	2010 to 2017	36	46	59.0	38/8	Yes	Yes
Kilic [29]	USA	CS	IV	2011 to 2021	58	77	57.3	49/28	Yes	Yes
Kim [30]	South Korea	RC	III	2008 to 2013	24	62	58.4	42/20	No	No
Lafosse [31]	France	CS	IV	2000 to 2002	29	17	47.0	13/4	No	No
Nove-Josserand [32]	France	RC	IV	2004 to 2007	36	22	54.7	19/3	No	No
Oklaz EB., 2024 [33]	Turkey	CS	IV	2017 to 2022	43	20	48.5	10/10	No	No
Rhee [34]	Republic of Korea	CS	IV	2005 to 2013	49	45	56.2	28/17	No	No
Seppel [35]	Germany	CS	IV	2004 to 2008	98	17	45.6	12/5	Yes	No
Yoon [11]	Republic of Korea	RC	III	2011 to 2016	24	56	57.1	32/24	No	No

COI, conflict of interest; CS, case series; PC, prospective cohort; RC, retrospective cohort

### Characteristics of subscapularis tears and arthroscopic repair techniques

The included studies demonstrated substantial variability in the tear classification systems, surgical techniques, and eligibility criteria for arthroscopic repair of isolated SSC tears. Tear severity was most commonly assessed using the Lafosse classification system, although several studies used alternative systems, such as the Fox and Romeo or Yoo classifications. Most included patients had full-thickness tears corresponding to Lafosse types II–IV, whereas partial tears and massive tears with concomitant rotator cuff involvement were commonly excluded. Fatty infiltration was predominantly evaluated using the Goutallier classification adapted by Fuchs et al., with most studies including patients with mild-to-moderate fatty degeneration and excluding advanced fatty infiltration (grade III–IV or higher).

Arthroscopic repair was primarily performed using single-row suture anchor techniques; however, several studies have reported modified or mixed techniques, including double-row, pseudo-double-row, suture-bridge, or Mason–Allen configurations, depending on the tear size and tendon mobility. Suture configurations commonly included mattress stitches, lasso-loop stitches, and modified Mason–Allen constructs using double-loaded anchors with nonabsorbable sutures. Concomitant long head of the biceps tendon procedures, particularly tenotomy or tenodesis, were frequently performed in the studies.

Surgical indications were generally based on symptomatic isolated SSC tears associated with pain, weakness, functional limitations, or failed conservative management. Most studies excluded patients with concomitant supraspinatus or infraspinatus tears, prior shoulder surgery, advanced osteoarthritis, inflammatory arthropathy, and severe fatty infiltration. Several studies required a minimum follow-up duration of 12–24 months and confirmation of isolated full-thickness SSC tears using arthroscopy or MRI.

Across all included studies, concomitant pathology of the long head of the biceps tendon (LHBT) was consistently observed in most patients undergoing arthroscopic or open repair of isolated SSC tears, with reported incidences ranging from 61 to 93%. The most frequently observed LHBT pathologies included subluxation or dislocation due to disruption of the biceps pulley system, partial tears, tenosynovitis, and SLAP lesions. In response to these findings, most surgeons performed concomitant biceps tenodesis (55.6–100% of cases across studies) or, less commonly, tenotomy. These data confirm that LHBT pathology is not an exclusion criterion for isolated SSC tear studies, as the biceps tendon is anatomically distinct from the rotator cuff. Rather, the high prevalence reflects the close anatomical relationship between the subscapularis tendon and the biceps pulley mechanism, where subscapularis tears frequently lead to medial wall disruption and subsequent biceps instability. More details were presented in Additional Table A1.

### GRADE certainty of evidence

Most outcomes had low to very low GRADE certainty of evidence. No result obtained high or moderate certainty of evidence, mainly due to the predominance of case series and retrospective cohort studies, which represented 64.7% Level IV evidence. Low-certainty evidence was found for five functional effect estimates: ASES score (MD 48.1, 95% CI 46.8 to 49.4), SSV (MD 48.2%, 95% CI 46.7 to 49.7), UCLA Shoulder Score (MD 15.4, 95% CI 14.8 to 15.9), external rotation (MD 5.2°, 95% CI 2.1 to 8.3), and internal rotation function effects. The remaining outcomes, including Constant-Murley Score, VAS pain, clinical tests, and strength tests, showed low or very low-certainty evidence. The most frequent reasons for downgrading were serious risk of bias for observational studies, very serious inconsistency (I2 > 90% for several outcomes), and suspected publication bias. Sensitivity analyses revealed similar results (Additional Table A2).

### Risk of bias assessment

**Fig. 2:**
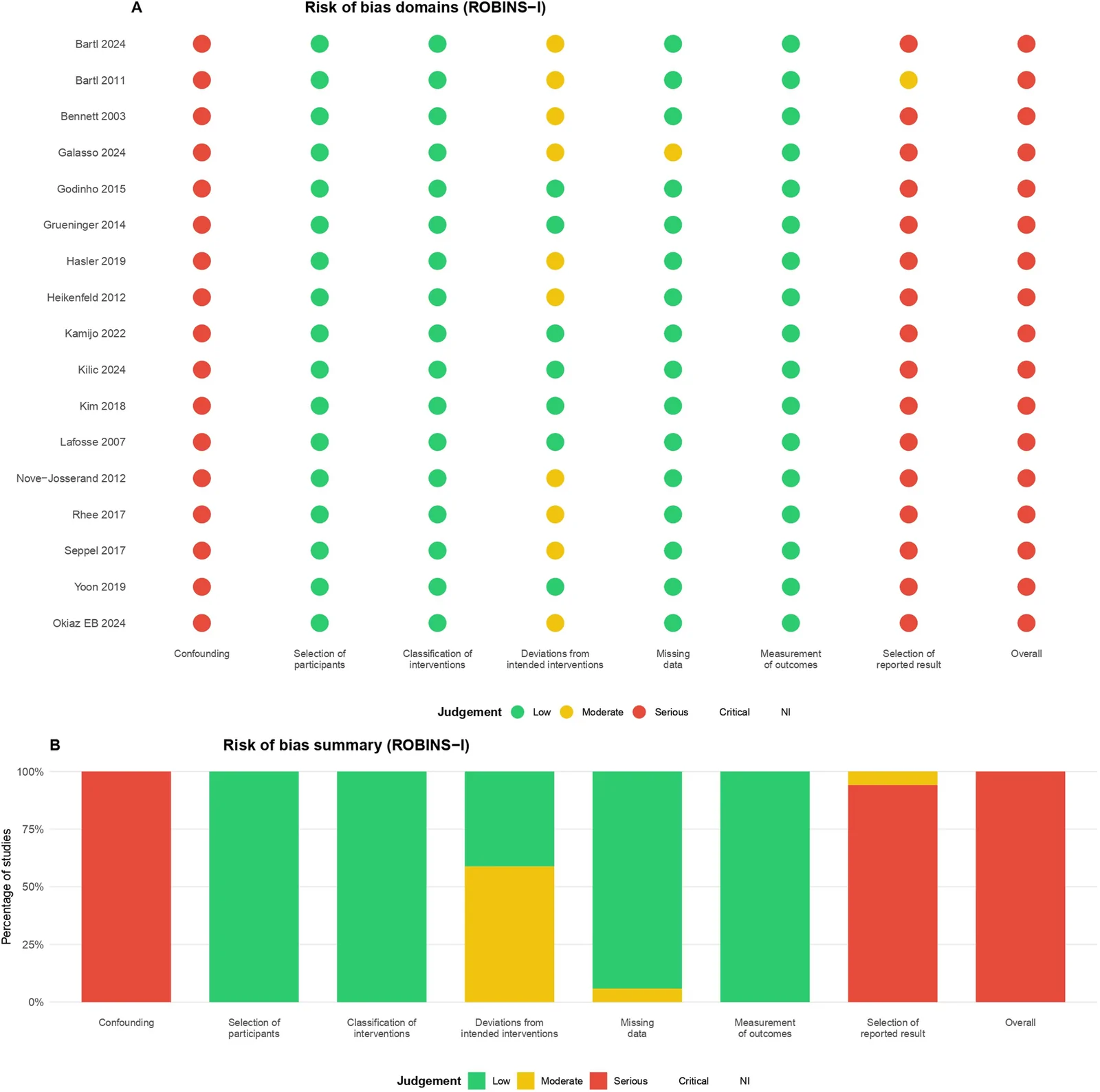
Risk of bias assessment using the ROBINS-I tool

Risk of bias was assessed for 17 non-randomized studies using the ROBINS-I tool (Fig. 2). Overall, 17 studies (100%) were judged to be at serious risk of bias. Regarding individual domains, the most common source of bias was confounding, with 17 studies (100%) rated at serious risk due to the non-randomized study design. Selection of participants was generally well-handled, with 17 studies (100%) at low risk. Classification of interventions showed low risk of bias across all studies. For deviations from intended interventions, 7 studies were at low risk and 10 at moderate risk. Missing data and measurement of outcomes were generally at low risk across included studies. Selection of reported results showed serious risk in most studies due to potential selective reporting concerns.

### Functional outcome scores

**Fig. 3:**
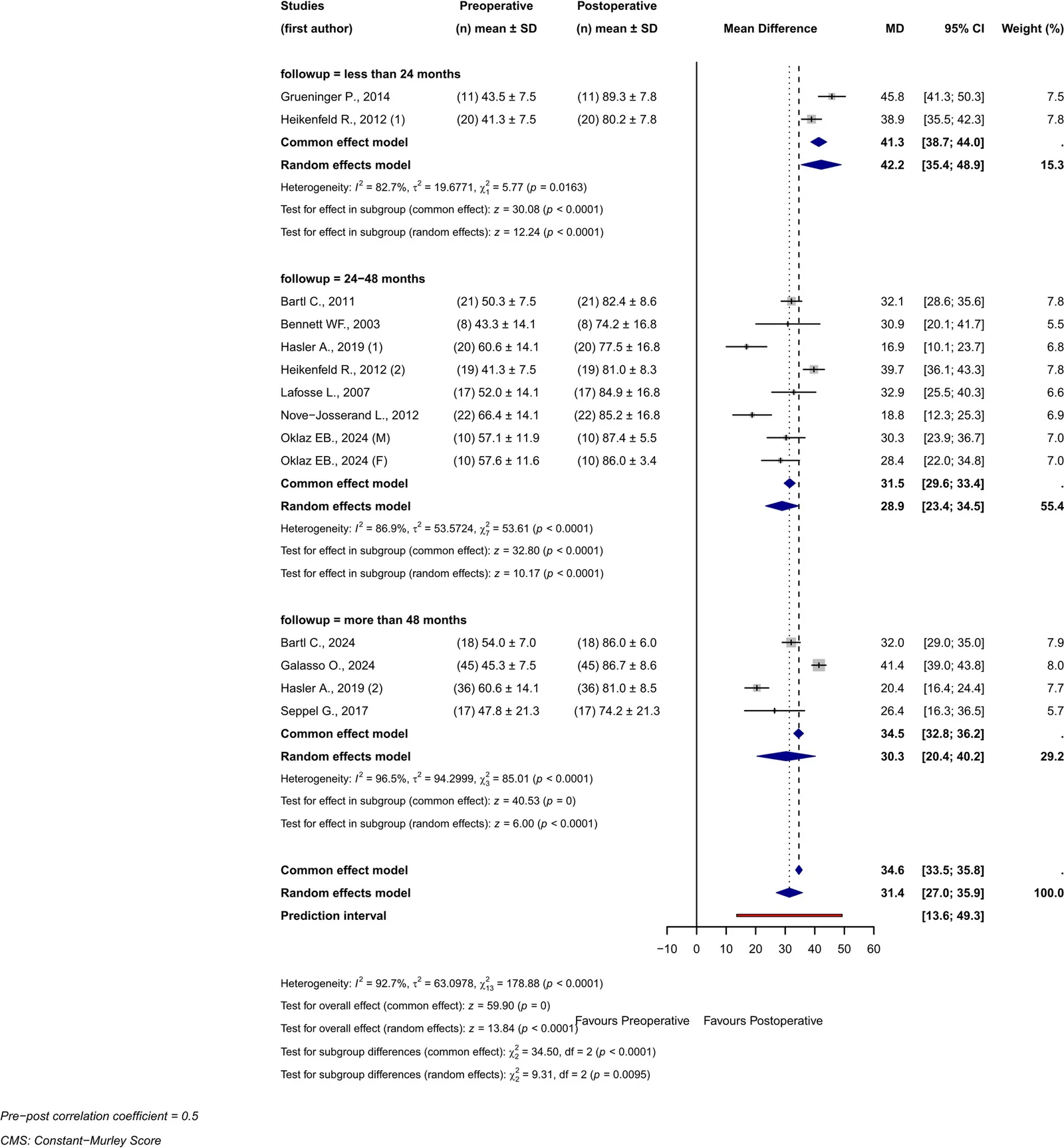
Forest plot of the mean difference in constant-murley score (CMS) from preoperative to postoperative, with subgroup analysis by follow-up duration

**Fig. 4:**
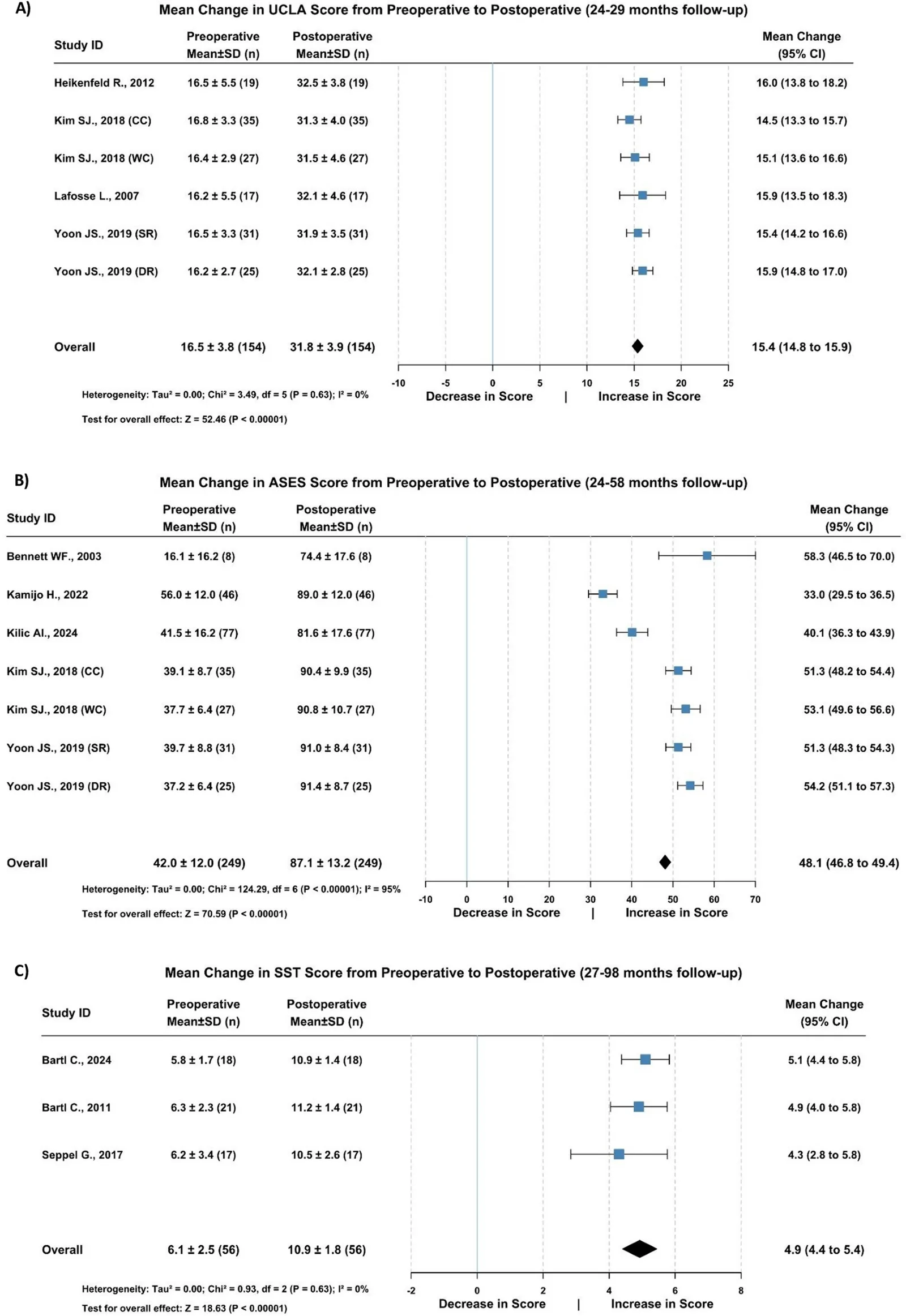
Forest plots of the mean change from preoperative to postoperative for: **A** University of California at Los Angeles Score (UCLA), **B** American Shoulder and Elbow Surgeons Score (ASES), and **C** Simple Shoulder Test (SST). CC, concomitant coracoplasty; WC, without coracoplasty; DR, double-row repair; SR, single-row repair

All 17 studies reported improvements in mean functional outcome scores following arthroscopic repair at the study level. Individual study effects varied substantially across outcomes: Preoperative-to-postoperative improvements in CMS ranged from 16.9 to 45.8 points (pooled MD = 31.4, 95% CI [27.0–35.9], *P* < 0.0001; I2 = 92.7%, *P* < 0.0001) (Fig. 3), UCLA gains varied from 14.5 to 16.0 points (pooled MD = 15.37, 95% CI [14.80–15.94], *P* < 0.00001; I2 = 0.0%, *P* = 0.63) (Fig. 4A), ASES improvements ranged from 33.0 to 58.3 points (pooled MD = 48.09, 95% CI [46.76–49.43], *P* < 0.00001; I2 = 95.2%, *P* < 0.00001) (Fig. 4B), SST gains varied from 4.3 to 5.1 points (pooled MD = 4.93, 95% CI [4.41–5.45], *P* < 0.00001; I2 = 0.0%, *P* = 0.63) (Fig. 4C), and SSV showed consistent improvements (pooled MD = 48.20, 95% CI [46.65–49.75], *P* < 0.00001; I2 = 39%, *P* = 0.15) (Additional Fig. A1A).

The exploratory subgroup analysis of CMS by follow-up duration revealed varying effect sizes between groups (*P* < 0.0001), though interpretation is limited by high heterogeneity. Individual studies consistently reported improvements in all time periods, with the highest individual effect in the < 24 months follow-up subgroup (individual effects ranging 38.9 to 45.8 points; pooled MD = 42.2, 95% CI [35.4–48.9], *P* < 0.0001; I2 = 82.7%, *P* = 0.0001), compared with 24–48 months subgroup (individual effects ranging 16.9 to 39.7 points; pooled MD = 28.9, 95% CI [23.4–34.5], *P* < 0.0001; I2 = 86.9%, *P* < 0.0001) and the > 48 months subgroup (individual effects ranging 20.4 to 41.4 points; pooled MD = 30.3, 95% CI [20.4–40.2], *P* < 0.0001; I2 = 96.5%, *P* < 0.0001), as shown in Fig. 3.

Funnel plot analysis for all functional scores showed no evidence of publication bias as shown in Additional Fig. A2. The leave-one-out sensitivity analyses confirmed that these results were robust (Additional Table A3).

The range of individual study effects and substantial heterogeneity across outcomes suggest that while consistent direction of benefit is observed across studies, the magnitude of clinical improvement varies considerably between patient populations and surgical contexts.

### Pain outcomes

A pooled analysis of five studies, as summarized in Additional Table A4, reporting the VAS for pain, demonstrated a significant postoperative reduction (MD = − 5.03, 95% CI [− 5.22, − 4.83], *P* < 0.00001; I2 = 91.3%, *P* < 0.00001) (Additional Fig. A1B). The funnel plot suggested a potential for publication bias (Additional Fig. A3A). Leave-one-out sensitivity analysis confirmed that the overall finding of significant pain relief remained robust (Additional Table A3).

### Subscapularis-specific clinical tests

Postoperative assessment revealed a significant reduction in the rate of positive findings across all subscapularis-specific clinical tests (Additional Table A5). The pooled analysis of nine studies showed a significant decrease in the risk of a positive lift-off test (RR = 0.19, 95% CI [0.14, 0.27], *P* < 0.00001; I2 = 0.4%, *P* = 0.11), as shown in Additional Fig. A4A. Similarly, an analysis of eight studies for the belly-press test (RR = 0.36, 95% CI [0.29, 0.46], *P* < 0.00001; I2 = 0.7%, *P* = 0.001) (Additional Fig. A4B), and three studies for the bear-hug test (RR = 0.46, 95% CI [0.35, 0.61], *P* < 0.00001; I2 = 0.6%, *P* = 0.09) also found significant reductions (Additional Fig. A4C). These results showed consistent direction of effect across studies, with negligible statistical heterogeneity (I2 < 1% for all three tests).

Funnel plots suggested a potential for publication bias for the lift-off and belly-press tests (Additional Fig. A3) but showed no evidence of publication bias for the bear-hug test (Additional Figure A5). Leave-one-out analyses confirmed the stability of these findings (Additional Table A3).

### Range of motion (ROM)

Arthroscopic repair of isolated subscapularis tendon tears resulted in significant postoperative improvements in range of motion (Additional Table A5). Internal rotation improved significantly on both measurement scales: by the Constant-Murley scale (MD = 2.38, 95% CI [2.03, 2.74], *P* < 0.00001; I2 = 80.9%, *P* < 0.00001) as represented in Additional Fig. S6, and by the spinal level scale (MD = − 3.45, 95% CI [− 3.74, -3.16], *P* < 0.00001; I2 = 76.4%, *P* < 0.00001), as shown in Additional Fig. S7. External rotation also showed a modest but significant gain (MD = 5.2, 95% CI [2.1, 8.3], *P* = 0.001; I2 = 80.0%) (Additional Fig. A8).

Despite substantial heterogeneity across analyses, funnel plots for internal rotation scales showed a low risk of publication bias (Additional Fig. A5). Leave-one-out sensitivity analyses confirmed that the overall conclusions for all ROM outcomes were robust (Additional Table A3).

### Strength outcomes

Postoperative strength of the SSC muscle showed significant improvements across both measured tests (Additional Table A5). Belly press strength demonstrated a significant mean increase (MD = 1.11, 95% CI [1.03, 1.19], *P* < 0.00001; I2 = 96.5%) (Additional Fig. A9). Similarly, lift-off strength showed a significant improvement (MD = 1.8, 95% CI [1.6, 2.0], *P* < 0.00001; I2 = 0%, *P* = 0.33) (Figure A10). The very high statistical heterogeneity observed for belly press strength and the potential publication bias suggested by its funnel plot (Additional Fig. A3) warrant cautious interpretation; in contrast, the analysis of lift-off strength showed negligible heterogeneity (I2 = 0%).

## Discussion

### Summary of key findings

In this systematic review with exploratory meta-analysis of non-randomized studies, we synthesized preoperative and postoperative data from 17 studies comprising 537 patients who underwent arthroscopic repair for isolated SSC tears. Pooled estimates suggest that arthroscopic repair was associated with postoperative improvements in functional outcome scores (CMS, ASES, UCLA, SST, and SSV), pain (VAS), range of motion, and a reduction in positive subscapularis-specific clinical tests (lift-off, belly-press, and bear-hug). However, these findings should be interpreted in light of the low to very low certainty of evidence (GRADE) and the serious risk of bias of all included studies (ROBINS-I), which preclude causal inferences about treatment effectiveness. The CMS, ASES, and UCLA clinical scales reflect improvements in postoperative outcomes, including reductions in pain and gains in shoulder function, ROM, strength, and ability to perform activities of daily living. The exploratory quantitative synthesis suggests statistically significant improvements across all assessed outcomes; however, the observational design of the available evidence and variability in outcome reporting warrant cautious interpretation regarding the clinical magnitude of these effects.

The observed improvements exceeded the previously reported MCID thresholds for outcomes for which published thresholds were available. Specifically, the MCID values were as follows: Constant Score 10.4 points [36]; ASES 10.2–27.1 points [37–39]; SST 4.3 points [38]; VAS pain 1.2–2.4 points [37–39]; and UCLA 6.0 points [37]. These consistent exceedances suggest clinically relevant patient benefits that extend beyond statistical significance. However, these MCID thresholds were derived from heterogeneous rotator cuff tear populations, limiting their direct applicability to isolated subscapularis repair. MCID interpretation was not applied uniformly to all outcomes because validated thresholds are not available for every score, ROM measure, strength assessment, or clinical test in isolated SSC tears. The moderate-to-high heterogeneity observed (I2 = 46–89%) further underscores this variability and constrains generalizability. Statistically and clinically relevant improvements were also observed in the SST, SSV, and VAS [40], reflecting improvements in patient-reported shoulder function, perceived shoulder capacity, and pain reduction, respectively; however, substantial heterogeneity was noted particularly in VAS outcomes. Subscapularis-specific clinical tests (lift-off, belly-press, and bear-hug) demonstrated a significant reduction in positivity following repair, with low heterogeneity and high consistency across studies. Internal rotation mobility, most often assessed using clinical ROM examination rather than instrument-based measurement, showed functional recovery supported by sensitivity analyses. Muscle strength also increased significantly, with the lift-off position showing the greatest gain and lowest variability, suggesting improved functional restoration of the subscapularis after arthroscopic repair.

A key finding from our subgroup analysis of the CMS outcome revealed that although these benefits were maintained long-term, the greatest magnitude of improvement occurred within the first two years post-surgery. Moreover, while the reduction in positive subscapularis-specific tests suggests resolution of insufficiency in most patients, a subset of patients in the included studies continued to demonstrate persistent test positivity. These findings indicate that complete restoration of the subscapularis function was not achieved in all cases. This may reflect factors such as baseline tendon quality, fatty infiltration, chronicity of tears, and variability in healing potential, although the included studies did not consistently report sufficient data to determine the exact underlying cause. Finally, the interpretation of these otherwise strong findings should be approached with caution. Funnel plot analysis suggested the potential for publication bias in some outcomes, particularly pain and subscapularis-specific clinical tests, which may indicate a slight overestimation of the true treatment effect.

### Interpretation of key findings

The pooled improvements in functional scores and reduction in pain observed across studies were associated with the arthroscopic repair of isolated subscapularis tendon tears. However, in the absence of a control group and given the low-to-very-low certainty of evidence, these associations should not be interpreted as causal effects. The findings should also be interpreted in the context of variability in surgical techniques (including repair configuration and concomitant procedures), postoperative rehabilitation protocols, baseline tendon pathology, and the substantial heterogeneity observed across most pooled outcomes.

The mechanistic basis for this improvement lies in the re-establishment of the transverse force couple of the shoulder [26, 41]. The SSC is the dominant internal rotator and a critical horizontal and vertical stabilizer [28]. Its repair restores the force balance, preventing the anterior subluxation of the humeral head that often causes pain and dysfunction [25, 42]. This restored stability allows for smoother glenohumeral kinematics, which is reflected in the broad functional gains captured by the various scoring systems. This is supported by early cohort studies, such as those by Lafosse et al. [31] and Heikenfeld et al. [27], which reported that arthroscopic repair was associated with favorable functional outcomes. Furthermore, the long-term studies by Hasler et al. [26] and Galasso et al. [23], confirmed that these gains are durable for nearly a decade post-surgery. This durability is further characterized by our subgroup analysis, which revealed that, although the greatest magnitude of improvement occurs within the first two years as the tendon heals and neuromuscular pathways adapt, these clinical benefits are well-maintained thereafter without significant late deterioration. Our findings are therefore consistent with this body of evidence, supporting an association between arthroscopic repair and postoperative improvement in this patient population.

The observed reduction in positive subscapularis-specific clinical tests postoperatively may reflect improved subscapularis tendon function following arthroscopic repair. However, these findings should be interpreted cautiously, as concomitant procedures, particularly long head of the biceps tenotomy or tenodesis, were frequently performed and may also have contributed to postoperative clinical improvement. [5, 28, 32, 34] However, the incomplete correction despite a structurally intact repair points to a critical disconnect between tendon integrity and full neuromuscular function. This may be attributed to the irreversible pathological changes within the SSC muscle itself. A strong correlation between a positive postoperative belly-press test and atrophy of the upper SSC muscle portion has been demonstrated by Bartl et al. [5] Persistent positive tests have also been associated with cranial SSC atrophy by Seppel et al. [35] Furthermore, a progression of fatty infiltration was observed in over 50% of patients post-repair in the study of Nové-Josserand et al. [32] Because fatty infiltration is widely considered irreversible, it leads to a permanent loss of contractile muscle fibers. Therefore, while surgery reattaches the tendon, it cannot reverse pre-existing muscle degeneration. This persistent muscle pathology may contribute to residual weakness or incomplete restoration of subscapularis function, which could explain the continued positivity of some subscapularis-specific clinical tests in a subset of patients.

The significant postoperative gain in internal rotation is an expected outcome, as the procedure directly restores the function of the primary internal rotator of the shoulder. [29] More intriguing is the modest but significant improvement in external rotation. This may be attributed to several procedural and physiological factors. First, the surgical technique itself often necessitates the release of the anterior capsule and rotator interval to achieve adequate mobilization of the retracted SSC tendon. This release of contracted soft tissues would mechanically increase the available passive external rotation [25]. Second, the resolution of preoperative pain and muscular guarding following a successful repair allows for a more physiological and less restricted range of motion in all planes [31]. Thus, the improvement in external rotation is likely multifactorial, reflecting the combined effects of surgical restoration, reduction of pain-related stiffness, and postoperative rehabilitation and physical therapy.

The significant postoperative increase in SSC strength may be associated with the restoration of the musculotendinous unit [5, 25]. By anatomically reattaching the SSC tendon to its footprint, arthroscopic repair re-establishes the continuity between muscle and bone, allowing the muscle to generate the internal rotation torque required to perform the lift-off, belly-press, and bear-hug maneuvers [31]. This allows the muscle to effectively generate the internal rotation torque required to perform the lift-off, belly-press, and bear-hug maneuvers. However, a critical aspect of our findings is the persistence of positive tests in some patients despite successful repairs. This may reflect multiple contributing factors, including baseline muscle quality, chronicity of the tear, surgical technique variability, rehabilitation differences, or incomplete functional recovery at the reported follow-up duration. Bartl et al. [5] linked a positive postoperative belly-press test to atrophy of the upper SSC on MRI, corroborated by Seppel et al. [35], in patients with cranial SSC atrophy. Nové-Josserand et al. [32] further documented postoperative progression of fatty infiltration in over half of their cohort, which irreversibly reduces the muscle’s force-generating capacity. Thus, our finding of incomplete correction suggests a pre-existing or progressive muscle degeneration, not surgical failure.

### Strengths and limitations

The primary strength of this meta-analysis is its comprehensive scope, representing the largest and most up-to-date synthesis of evidence on the clinical and functional outcomes of arthroscopic repair of isolated subscapularis tendon tears. Compared with previous systematic reviews, the present study included a larger pooled patient population and incorporated more recently published studies. In addition, we performed quantitative pooled effect analyses for multiple clinical and functional outcomes, including CMS, ASES, UCLA, SST, SSV, pain scores, range of motion, and strength-related subscapularis clinical tests. We also conducted subgroup analyses according to follow-up duration and performed a formal certainty-of-evidence assessment using the GRADE methodology. Collectively, these additions provide a broader and more clinically interpretable evaluation of postoperative outcomes following arthroscopic repair of isolated subscapularis tears.

By strictly including only isolated SSC tears, we aimed to enhance the clinical homogeneity of the study population compared with previous reviews that included mixed rotator cuff tears. However, substantial statistical heterogeneity was still observed across most pooled outcomes, reflecting variability in patient characteristics, tear morphology, surgical techniques, and outcome assessment methods across studies. Furthermore, the subgroup analysis by follow-up duration provides crucial insights into the temporal dynamics of functional recovery. All included studies were judged to be at serious risk of bias using the ROBINS-I tool, and the certainty of the evidence was rated as low or very low for all outcomes using the GRADE approach. These findings limit the strength of the conclusions drawn. Nevertheless, the leave-one-out sensitivity analyses confirmed that the pooled estimates were not unduly influenced by any single study, supporting the internal stability of the results within the limitations of the evidence base.

Several important limitations warrant emphasis. First, the analysis is based entirely on non-randomized studies, which carry inherent risks of selection bias and confounding, limiting causal inferences about treatment effects. Second, although the broad 2000–2025 search window was necessary to maximize available evidence for this uncommon injury pattern, advances in imaging, arthroscopic instrumentation, fixation methods, and rehabilitation protocols across this period may have introduced clinical and methodological heterogeneity. Third, substantial heterogeneity across outcomes (I2 > 80% in most analyses) may reflect differences in study design, patient selection, tear severity, surgical techniques, rehabilitation protocols, follow-up duration, and outcome assessment methods among the included studies. Although additional subgroup analyses or meta-regression could theoretically help explore potential effect modifiers, these analyses were not feasible because of the limited number of studies available for several outcomes and inconsistent reporting of relevant covariates across studies. Performing meta-regression under these conditions can produce unstable or unreliable estimates. Therefore, random-effects models were applied throughout the analyses, and subgroup analysis according to follow-up duration was performed for the primary outcome where sufficient data were available. The observational design precludes definitive conclusions about treatment efficacy. Fourth, another major limitation of this meta-analysis is the frequent co-performance of concomitant long head of the biceps tendon (LHBT) procedures, particularly tenotomy or tenodesis, which were performed in 55.6–100% of cases across the included studies. None of the studies provided consistent comparative analyses between isolated SSC repair and SSC repair combined with biceps tendon intervention. Consequently, the independent contribution of biceps procedures to postoperative improvement could not be determined, and the observed improvements in pain, function, and clinical tests cannot be attributed to subscapularis repair alone.

Furthermore, the assumed pre-post correlation coefficient of 0.5 used to calculate change-from-baseline standard deviations was not tested under alternative values, which may affect the magnitude of the pooled estimates and represents a further methodological limitation of the present analysis.

Additionally, persistent postoperative strength deficits were observed in a subset of patients, but the included studies did not consistently report or stratify outcomes according to preoperative prognostic factors such as Goutallier fatty infiltration stage or tendon retraction severity. Therefore, it was not possible to perform a meta-analytic assessment of predictors of incomplete strength recovery. Nevertheless, existing literature suggests that higher degrees of fatty infiltration and chronic tendon degeneration may negatively influence postoperative functional recovery.

Finally, potential publication bias was detected for some outcomes, suggesting that the true treatment effect may have been slightly overestimated. In addition, the overall certainty of evidence was limited by the predominantly observational design of the included studies, and all studies demonstrated a high risk of bias, which should be considered when interpreting the pooled findings.

### Clinical implications and future research

This meta-analysis synthesizes the currently available observational evidence on postoperative outcomes following arthroscopic repair of isolated SSC tears. In the included studies, arthroscopic repair was associated with postoperative improvements in shoulder function and pain, supporting its role as a treatment option for these conditions. However, given the low to very low certainty of evidence, these findings should be used to counsel patients with appropriate caution regarding the magnitude and durability of the expected benefits. The key clinical implication therefore lies in appropriately managing patient expectations regarding postoperative recovery, particularly with respect to pain improvement, strength restoration, and the potential for persistent functional deficits despite overall clinical improvement. Our findings show that a persistent, quantifiable strength deficit often remains despite a structurally intact repair. This suggests that the primary determinant of postoperative strength is the preoperative health of the SSC muscle itself. Therefore, this analysis provides a strong rationale for advocating for early diagnosis and intervention in symptomatic patients to mitigate the onset of irreversible muscle atrophy and fatty infiltration, thereby maximizing the potential for a more complete functional recovery.

While these findings guide current practice, they also highlight critical evidence gaps that must be addressed by future research. The predominance of non-randomized data in our analysis underscores the need for large-scale, adequately powered RCTs to definitively confirm these outcomes and to compare different repair techniques (e.g., single- versus double-row). Future trials should mandate the use of objective, quantitative strength measurement and standardized MRI protocols to prospectively track the relationship between muscle degeneration and functional recovery. This would allow for a more precise identification of the prognostic threshold at which fatty infiltration becomes irreversible. Future meta-analyses should also explore the sensitivity of pooled pre-post estimates to alternative assumed correlations (e.g., 0.3 and 0.7) to confirm the robustness of the findings. Finally, given that concomitant biceps procedures are a major confounder, dedicated studies are needed to better isolate the effects of the SSC repair, potentially by stratifying outcomes based on the type of biceps intervention performed. To capture the long-term durability of these repairs, all future investigations must incorporate a pre-specified long-term follow-up.

### Conclusion

Individual studies have consistently reported postoperative improvements in clinical function, pain, and range of motion after arthroscopic repair of isolated SSC tears. However, these findings are based on non-randomized studies with a serious risk of bias, low to very low certainty of evidence (GRADE) for all outcomes, substantial statistical heterogeneity, and potential publication bias for some outcomes. In addition, the frequent co-performance of long head of the biceps tenotomy or tenodesis represents a major confounder that limits the ability to attribute the observed improvements to subscapularis repair. Available data also suggest that postoperative strength deficits may persist in a subset of patients, likely reflecting pre-existing muscle pathology, such as atrophy and fatty infiltration rather than surgical failure.

These findings highlight the importance of early diagnosis and timely intervention to preserve muscle quality and optimize functional recovery, particularly in terms of strength outcomes. Further high-quality prospective studies and randomized trials are needed to better compare repair techniques and to isolate the effect of arthroscopic SSC repair from that of concomitant long head of the biceps procedures, and to investigate strategies aimed at mitigating muscle degeneration and improving long-term functional strength.

## Supplementary Information

Below is the link to the electronic supplementary material.

Supplementary file 1

## Data Availability

All data analyzed in this study are derived from published articles included in the systematic review. The datasets generated and/or analyzed during the current study are available from the corresponding author upon reasonable request.
