# Supplementary material for: Postoperative outcomes after arthroscopic repair of isolated subscapularis tears: a systematic review and meta-analysis
Source: J Orthop Surg Res. 2026 Jul 17;21:590. doi: 10.1186/s13018-026-07088-5 (PMC13660073; doi:10.1186/s13018-026-07088-5)
Supplement: Supplementary file 1 [file 13018_2026_7088_MOESM1_ESM.docx]

**Additional Files, Tables, and Figures**

**Additional Files**

**Additional File 1.** Research strategy on different data bases.

("subscapularis tears"[Title/Abstract] OR "subscapularis tear"[Title/Abstract] OR "subscapularis tendon"[Title/Abstract] OR "subscapularis injury"[Title/Abstract] OR "subscapularis injuries"[Title/Abstract] OR subscapularis lesion"[Title/Abstract]) AND isolated[Title/Abstract] AND arthroscop*[Title/Abstract].

**PubMed Search Strategy**

(“subscapularis tear”[Title/Abstract] OR “subscapularis tears”[Title/Abstract] OR “subscapularis tendon”[Title/Abstract] OR “subscapularis injury”[Title/Abstract] OR “subscapularis injuries”[Title/Abstract] OR “subscapularis lesion”[Title/Abstract])

AND

isolated[Title/Abstract]

AND

arthroscop*[Title/Abstract]

**Scopus Search Strategy**

TITLE-ABS-KEY (“subscapularis tear” OR “subscapularis tears” OR “subscapularis tendon” OR “subscapularis injury” OR “subscapularis injuries” OR “subscapularis lesion”)

AND TITLE-ABS-KEY (isolated)

AND TITLE-ABS-KEY (arthroscop*)

**Cochrane Library Search Strategy**

(“subscapularis tear” OR “subscapularis tears” OR “subscapularis tendon” OR “subscapularis injury” OR “subscapularis injuries” OR “subscapularis lesion”):ti,ab,kw

AND isolated:ti,ab,kw

AND arthroscop*:ti,ab,kw

**Additional Tables**

**Additional Table A1. Baseline characteristics of the included patients**

| **Study ID** | **Classification system used** | **Distribution of tear severity** | **Surgical technique details (single-row vs. double-row)** | **Suture configuration** | **Tear size** | **Fatty infiltration grade** | **Surgical indications or inclusion criteria for arthroscopic repair** | **Reported Incidence of LHBT Pathology** | **Specific Findings** |
| --- | --- | --- | --- | --- | --- | --- | --- | --- | --- |
| Bartl C., 2024^22^ | Lafosse classification (for subscapularis tear size). Fuchs et al. MRI grading system based on adapted Goutallier CT score (for fatty infiltration grade). Sugaya classification (for postoperative tendon repair integrity). | Arthroscopic Repair Group (ARG): Type II: 5 Type III: 7 Type IV: 6  Open Repair Group (ORG): Type II: 5 Type III: 7 Type IV: 4 Note: Type I (partial) and Type V (with subluxation/combined tears) were excluded. | Single-row suture anchor repair technique (used uniformly in both groups). | Bioabsorbable suture anchors (Bio-Corkscrew® 5.5mm; Arthrex), double-loaded with non-absorbable sutures (Fiberwire®; Arthrex). Knots were tied after perforation with a suture passer. | NR | Preoperative (ARG): Grade 0 & 1 in 89%, Grade 2 in 11% Postoperative (ARG): Grade 0 & 1 in 83%, Grade 2 in 16%  Preoperative (ORG): Grade 0 & 1 in 81%, Grade 2 in 19% Postoperative (ORG): Grade 0 & 1 in 75%, Grade 2 in 25% | Isolated full-thickness subscapularis tendon tear confirmed at diagnostic arthroscopy. Patients with arthroscopic repair (ARG) were part of a prospective non-randomized study at two centers. Exclusion criteria for both groups: partial articular-sided subscapularis tears, tears with involvement of supraspinatus/infraspinatus, Lafosse Type I (partial) and Type V (humeral head subluxation with combined tears). | 89% (16/18) in arthroscopic group; 93% (15/16) in open group | Lesion with subluxation or dislocation out of bicipital groove. Treated with tenodesis (13/18, 14/16) or tenotomy (3/18, 1/16). |
| Bartl C., 2011^5^ | Tear severity: Fox & Romeo classification (Type II, III, IV).  Tendon retraction: Grade 1, 2, or 3 (based on stump location).  Fatty infiltration: Fuchs et al. MRI grading system based on Goutallier CT score (FIG 0–4).  Cartilage lesions: Modified Outerbridge classification (grades 1–4). | By Fox & Romeo classification (arthroscopic group):  Type II (upper 25% of tendon): 4 shoulders  Type III (upper 50% of tendon): 10 shoulders  Type IV (>50% craniocaudal diameter): 7 shoulders   (Type I partial tears were excluded) | Single-row suture anchor repair (all cases). | Suture anchor (Titancorkscrew 5mm or Biocorkscrew 5mm; Arthrex), double-loaded with number 2 braided nonabsorbable sutures (Fiberwire; Arthrex). Knots tied with arm in slight abduction and 20° external rotation. | Average anchors used: 2.2 (range 1–4). | NR | Confirmed isolated full-thickness subscapularis tendon tear at arthroscopy.  Clinically symptomatic tear with persistent pain and weakness.  Excluded: Partial articular-sided tears (Fox type I), shoulders with other concomitant rotator cuff tears. | Not reported as overall % (but 16/21 had LHB treatment: 9 tenodesis, 1 tenotomy, 2 recentering) | Status: ruptured (2), subluxated (6), dislocated (3), partial tear (7), normal (6). |
| Bennett WF., 2003^23^ | - Biceps subluxation/instability: Author's own 5-type classification (Types 1–5) based on arthroscopic observation (involvement of subscapularis/IASS, medial head of coracohumeral ligament [MCHL], lateral head of coracohumeral ligament [LCHL], and supraspinatus). - Tear completion: Complete vs. partial IASS (intra-articular subscapularis) tears. | Total isolated subscapularis tears: 8 patients  - Complete IASS tears: 6  - Partial/incomplete IASS tears: 2   MCHL involvement:  - In complete tears: 4 patients (Type 3 biceps lesion)  - In partial tears: 1 patient | NR | Two sutures per screw (double-loaded). Sutures described as "Tevdek" (braided nonabsorbable). Knots: modified Revo knot, simple knots, and mattress knots. | NR | NR | - Tear of the subscapularis tendon (IASS) or medial head of the coracohumeral ligament.  - Exclusion criteria: involvement of any other rotator cuff tendon (including partial tears of supraspinatus), or any other intra-articular lesion found at arthroscopy.  - Minimum 2 years follow-up required for inclusion in the study cohort. | Not reported as overall % (8 patients, all with isolated tears) | Describes biceps subluxation/instability classification (Types 1-5). MCHL involved in 4/6 complete tears (Type 3 lesion). |
| Galasso O., 2024^24^ | - Tear severity: Lafosse classification (Types I–V).  - Tendon healing (postoperative): Sugaya classification adapted for ultrasound assessment (Types I–V).  - Fatty degeneration (indirectly for Type V): Goutallier stage (mentioned for Type V definition: > Goutallier stage III). | By Lafosse classification (included patients only):  - Type III (full-thickness tear in upper two-thirds): 28 patients (62.2%)  - Type IV (complete tear without tendon retraction): 17 patients (37.8%)   (Types I, II, and V were excluded per inclusion criteria) | Single-row technique. All repairs described as "watertight" at end of intervention. | Suture anchors with 3 strands per anchor (Fastin RC; DePuy Mitek or TwinfixTi; Smith & Nephew).  Stitch technique: modified lasso-loop stitch used in all cases. | NR | NR | - Primary and elective shoulder arthroscopy for isolated SSC repair. - Lafosse type III or type IV SSC tear. - Minimum 24-month follow-up. - Symptomatic patients of working age with preserved SSC muscle belly, negligible osteoarthritis, and absence of capsulitis.  Exclusion criteria: revision rotator cuff repair, prior shoulder surgery, fracture, dislocation, glenohumeral osteoarthritis, neurological disorders, cognitive impairment, inability to complete questionnaires. | Not reported as primary outcome (biceps tenotomy performed in 32/45, 71.1%) | Tenotomy performed before SSC repair. No specific incidence of biceps pathology given. |
| Godinho GG., 2015^25^ | - 3-type system for complete tears based on MRI (affecting upper third, upper two thirds, or total tear). - Not using Lafosse, Fox, or Fuchs/Goutallier classifications. - Lift-off and belly-press tests for subscapularis function. - Constant-Murley score with Boehm validation categories (excellent, good, satisfactory, adequate, weak). | - Upper third deinsertion: 3 (17%)  - Upper two thirds: 9 (50%)  - Complete deinsertion (total): 6 (33%)   (Partial thickness-only tears were excluded) | NR | NR | NR | NR | - Isolated tearing or deinsertion that was complete OR affected at least one third of the subscapularis tendon.  - Degenerative or traumatic origin.  - Associated with functional deficit and pain.   Exclusion criteria: follow-up <1 year, partial thickness-only tears (without complete deinsertion of part or all of tendon), any associated lesions in other rotator cuff tendons, open repair, rerupture after operation, sequelae of infection, advanced osteoarthritis. | 61% (11/18) | LHBT affected: dislocated/subluxated (8), torn (2), stable with >50% tear (1). Tenotomy (6), tenodesis (3). |
| Grueninger P., 2014^26^ | - Tear severity: Lafosse classification (specifically Types III and IV).  - Fatty infiltration: Goutallier et al. modified by Fuchs et al. for MRI (grades 0–4).  - Muscle cross-sectional area (CSA): Zanetti et al. method. | By Lafosse classification (N = 11):  - Type III (complete lesion of superior two-thirds): 9 patients (82%)  - Type IV (complete tear with retraction but no subluxation): 2 patients (18%)   (Types I, II, and V excluded per inclusion criteria) | Mixed technique:  - Type III lesions: "Pseudo-double-row" reconstruction (2 threaded suture anchors with U-stitches + one lasso-loop stitch at upper border using remaining suture from biceps tenodesis anchor).  - Type IV lesions with higher tension (n=2): Double-row reconstruction with suture-bridge technique.   Also notes: single-row repair for LHBT tenodesis only; no coracoplasties performed. | - U-stitches (for most anchors).  - Lasso-loop stitch at upper border of SSC.  - For Type IV with higher tension: double-row with suture-bridge technique.  - Biceps tenodesis: one suture of a double-loaded threaded suture anchor used for lasso-loop stitch. | NR | Preoperative (SSC): Mean 1.7 (range 0–3) | - Traumatic isolated complete SSC tendon tears.  - Lafosse type III or IV lesions only.  - Consecutive patients undergoing all-arthroscopic repair.   Exclusion criteria: Anterosuperior lesions, mass lesions, low-grade lesions (Lafosse I/II). | 91% (10/11) | LHBT completely dislocated (3), subluxated (5), anterior instability (2). Only 1 normal. All had anchor tenodesis. |
| Hasler A., 2019^27^ | - Tear severity: Lafosse classification (Grades I–IV).  - Fatty infiltration: Goutallier et al. for CT, adapted by Fuchs et al. for MRI (grades 0–4).  - Tendon/muscle lengths and musculotendinous junction location: Meyer et al. method. | By Lafosse classification (N = 36 shoulders):  - Grade I or II: 23 cases  - Grade III: 4 cases  - Grade IV: 9 cases | No double-row techniques used.  Single-row repair with suture anchors.  Number of anchors depended on tear size:  - Grade I or II: 1 anchor (mean 1.1)  - Grade III: 2 or 3 anchors (mean 2.3)  - Grade IV: 2 or 3 anchors (mean 2.6)   Overall mean anchors: 1.6 (range 1–4). | - Simple stitches or mattress stitches. - For most superior suture: a loop technique. - Stay suture (No. 1 polydioxanone) often used as traction for temporary reduction. - Shuttle technique with No. 1 polydioxanone if clever hook could not penetrate tendon. | NR | Subscapularis (mean, N=35 preop, N=31 postop):  - Preoperative: 0.48 (range 0–2)  - Long-term follow-up (mean 8.6 years): 1.3 (range 0–4)  - Significant progression (P < .001)   Supraspinatus: Preop 0.2 → Postop 0.8 (P < .001)  Infraspinatus: Preop 0.3 → Postop 0.8 (P = .002)   *Subgroup analysis (midterm to long-term): No significant progression between midterm (mean 2.9 years) and long-term (mean 8.6 years) for any muscle.* | - Patients of working age.  - Pain and impairment of shoulder function.  - Minimal osteoarthritis, no capsulitis.  - Fatty muscle infiltration ≤ Goutallier grade 2.   Exclusion criteria: Tears combined with supraspinatus or infraspinatus, fatty infiltration > Goutallier 2, bony avulsion, osteoarthritis (3 patients), additional procedures except biceps tenotomy/tenodesis, AC resection, or acromioplasty (2 patients excluded for this), prior shoulder surgery (2 patients), prior/subsequent proximal humeral fracture (3 patients), wheelchair dependence, preoperative capsulitis with substantial ROM loss, inflammatory arthropathy. | 69.4% (25/36) had tendinopathy; 13.9% (5/36) complete dislocation | Normal in 11 (30.6%). Tenotomy (20), tenodesis (5). |
| Heikenfeld R., 2012^28^ | - Tear severity: Lafosse classification (Types 2, 3, 4).  - Fatty infiltration: Goutallier et al. classification (stages 0–4).  - Osteoarthritis: Samilson and Prieto criteria. | By Lafosse classification (N = 20 patients):  - Type 2 (complete tear of upper one-third): 10 (50%)  - Type 3 (complete tear of upper two-thirds): 7 (35%)  - Type 4 (complete tear, humeral head centered): 3 (15%)   (Type 1 partial tears were excluded) | Single-row configuration.  One to three bone anchors, each loaded with 2 sutures.  Biceps tenodesis (when indicated) performed by lasso-loop technique using 1 suture of a double-loaded anchor placed proximal to bicipital groove; remaining suture used for subscapularis repair.  Final repair used arthroscopic Mason-Allen configuration. | - Each anchor loaded with 2 sutures.  - Mason-Allen configuration for final repair.  - For biceps tenodesis: lasso-loop stitch.  - Horizontal stitch performed with second suture when one suture used for tenodesis. | NR | By Goutallier classification (N = 20, preoperative MRI): - Stage 0 or 1: 17 tears (85%) - Stage 2: 3 tears (15%)  (No stages 3 or 4 reported)  Postoperative fatty infiltration not systematically reported for the cohort, though noted in failures: - Failure case 1: Goutallier stage 2 preop - Failure case 2: Goutallier stage 1 preop | - Isolated subscapularis tears treated with arthroscopic repair.   Exclusion criteria: Tears extending to supraspinatus tendon, partial tears (Lafosse type 1).   Note: Study included Lafosse types 2, 3, and 4 only. | 65% (13/20) | Concomitant pathology of LHBT (complete tear 1, partial tears/instability 12). Treated with tenodesis (12). |
| Kamijo H., 2022^29^ | - Tear severity: Lafosse classification (Types 1–5). - Fatty degeneration: Goutallier staging modified by Fuchs et al. (stages 1–4). - Postoperative repair integrity: Sugaya classification (Types 4 and 5 considered retears). | By Lafosse classification (N = 46 shoulders): - Type 1: 13 - Type 2: 10 - Type 3: 12 - Type 4: 6 - Type 5: 5  Grouped for analysis: - Smaller tears (Types 1–3): 35 shoulders - Larger tears (Types 4 and 5): 11 shoulders | Surgical technique details - Suture-bridging repair techniques used in most shoulders (41 of 46). - Single-row repair applied for some joint-side tears (Lafosse type 1; 5 shoulders). - For suture-bridging: medial anchors loaded with 3 high-strength sutures; lateral-row knotless anchors inserted in bicipital groove. - Tenodesis or tenotomy of long head of biceps performed in all patients. | - Single-row (Type 1 tears): Anchor loaded with 2 No. 2 high-strength sutures (HEALICOIL), passed through tendon and tied in mattress fashion. - Suture-bridging: Medial anchors with 3 sutures; suture-bridging done first using lateral-row anchors; remaining medial sutures tied in mattress fashion. - Comma fibers reduced with 2 suture limbs placed in rotator interval. | NR | By Goutallier/Fuchs stages (preoperative): - Smaller tears (Types 1–3): Stage 1 (13), Stage 2 (13), Stage 3 (8), Stage 4 (0) - Larger tears (Types 4–5): Stage 1 (0), Stage 2 (4), Stage 3 (2), Stage 4 (5)  Postoperative: - Smaller tears: Stage 1 (13), Stage 2 (14), Stage 3 (6), Stage 4 (1) - Larger tears: Stage 1 (1), Stage 2 (1), Stage 3 (2), Stage 4 (7)  Larger tears had significantly greater fatty degeneration both pre- and postoperatively (P < .001). No significant improvement in either group. | Surgical indications or inclusion criteria for arthroscopic repair Inclusion: Isolated subscapularis tear that underwent arthroscopic rotator cuff repair.  Exclusion criteria: (1) previous rotator cuff surgery, (2) lack of radiographic or clinical evaluation data, (3) neuromuscular diseases, (4) < 2-year follow-up. | Not directly reported (all had LHB tenotomy or tenodesis) | LHB treatment: tenodesis (26 in larger tears? — table shows 26 tenodesis, 6 tenotomy, 3 torn across groups). |
| Kilic AI., 2024^30^ | - Tear severity: Lafosse classification (Types I–V).  - Fatty infiltration: Goutallier classification (grades 0–4) for each rotator cuff muscle (upper and lower subscapularis, supraspinatus, infraspinatus).  - Subscapularis division for grading: Collin et al. method (upper vs. lower half). | By Lafosse classification (N = 77 patients):  - Type 1: 33 (44.2%)  - Type 2: 24 (29.9%)  - Type 3: 13 (16.9%)  - Type 4: 7 (9.1%)  - Type 5: 0 (0.0%)   Tear size as % cephalad to caudal (mean): 47.9% ± 25.6% | Both techniques used based on tear pattern and tendon mobility:  - Single-row: 54 patients (70.1%)  - Double-row: 23 patients (29.9%)   Biceps tenodesis performed in 69 patients (89.6%).  Coracoplasty performed if subcoracoid space <7 mm or coracoid spur present (24.7%).  Acromioplasty performed in 87%. | NR | Reported as percentage of cephalad to caudal tendon involvement: mean 47.9% ± 25.6%. | Preoperative Goutallier grades (N for each grade 0,1,2,3,4):  - Supraspinatus: 60, 9, 8, 0, 0  - Infraspinatus: 54, 14, 9, 0, 0  - Subscapularis upper part: 33, 21, 14, 4, 5  - Subscapularis lower part: 65, 8, 1, 2, 1   No postoperative fatty infiltration grades reported. | Inclusion: primary arthroscopic isolated subscapularis repair, baseline patient-reported outcomes, minimum 2-year follow-up.   Exclusion criteria: history of previous surgery, concomitant posterosuperior cuff repair, proximal humerus or glenoid fracture, inaccessible/poor quality MRI, incomplete physical examination or intraoperative documentation. | 70.1% (54/77) had biceps tenodesis; 7.8% (6/77) left intact | Intraoperative findings: intact (29.9%), subluxated/dislocated (18.2%), partial tear (41.6%), complete/retracted tear (10.4%). |
| Kim SJ., 2018^31^ | - Tear severity: Lafosse classification (Types II, III, IV).  - Fatty infiltration: Goutallier classification (Grade IV excluded).  - Osteoarthritis: Hamada classification (Grade ≥2 excluded).  - Coracohumeral distance measurement: Method described with parallel lines tangential to humeral head and coracoid. | By Lafosse classification (N = 62 patients): - Group A (with coracoplasty, n=35): Type II (7), Type III (24), Type IV (4) - Group B (without coracoplasty, n=27): Type II (5), Type III (19), Type IV (3)  (Types I and V not reported; full-thickness tears only) | Single-row repair in all cases.  Biceps tenotomy performed first in all patients; subsequent subpectoral biceps tenodesis in 22/35 (Group A) and 15/27 (Group B).  Coracoplasty (approx. 2 mm thick) performed in Group A using burr or acromionizer via superior portal. | NR | NR | Preoperative (Goutallier classification): - Grade IV fatty infiltration in subscapularis muscle was an exclusion criterion. - Distribution of grades I–III not reported numerically.  Both groups had similar preoperative CHD (~6.5–6.7 mm). | Inclusion: isolated subscapularis full-thickness tear, available for ≥2 years follow-up.   Exclusion criteria: previous surgery on affected shoulder, grade ≥2 glenohumeral osteoarthritis (Hamada), grade IV fatty infiltration of subscapularis (Goutallier), workers' compensation claim, unavailable for MRA/CTA at 6 months postoperatively.   Indication for surgery: pain and functional impairment refractory to ≥3 months non-operative treatment. | 86-89% (30/35 in Group A, 24/27 in Group B) | Biceps lesion with or without subluxation/dislocation. Subsequent tenodesis in 22 and 15 respectively. |
| Lafosse L., 2007^32^ | - Tear severity: 5-type classification (Types I–V) based on intraoperative evaluation and preoperative CT. - Fatty infiltration: Goutallier et al. classification (stages 0–4), simplified as minimal (0–1), intermediate (2), advanced (3–4). - Osteoarthritis: Samilson and Prieto criteria. | By authors' classification (N = 17 patients):  - Type I (partial lesion of superior third): 2  - Type II (complete lesion of superior third): 4  - Type III (complete lesion of superior two-thirds): 7  - Type IV (complete lesion, head centered, fatty degeneration ≤ stage 3): 4  - Type V (complete lesion, eccentric head, fatty degeneration ≥ stage 3): 0   (Also reported as: superior third = 7 patients, superior two-thirds = 6 patients, complete separation = 4 patients) | Mixed repair technique:  - For Type III and IV tears: mattress suture inferiorly/medially and simpler suture laterally in an attempt to perform a double-row suture anchor repair.  - Metallic anchors (G4 super anchor; Mitek) loaded with #2 Ethibond sutures.  - No coracoplasties performed.  - Biceps tenodesis in 9 of 15 unruptured biceps tendons with suture anchor fixation (G2; Mitek) using simple suture configuration. | - Mattress suture (inferiorly and medially) + simple suture (laterally over footprint) for Type III/IV tears.  - Shuttle-relay technique for passing sutures through tendon.  - Biceps tenodesis: simple suture configuration of #2 Ethibond. | NR | Preoperative (N = 17): - Stage 0 or 1 (minimal): 15 patients - Stage 2 (intermediate): 2 patients - Stage 3 or 4 (advanced): 0 patients  Postoperative: Progression of fatty infiltration was not observed in any patient. | - Symptomatic subscapularis tear with pain and weakness after traumatic injury, OR failure of nonoperative management in degenerative tears. - Isolated subscapularis tear (tears involving any other rotator cuff tendons excluded). - Type V tears (eccentric head with subcoracoid impingement) were not treated with arthroscopic repair in this series. | Not reported as overall % (2 ruptures, 6 normal, 2 mild fraying, 7 partial tear) | Nine of fifteen unruptured biceps tendons with pathologic changes underwent tenodesis. |
| Nove-Josserand L., 2012^33^ | - Tear size: By thirds of tendon (superior one-third, superior two-thirds, complete height) based on Lafosse et al. and Edwards et al.  - Fatty infiltration: Goutallier et al. classification modified by Fuchs et al. for MRI (stages 0–4).  - Tendon healing thickness: Adapted from Sugaya et al. (normal thickness vs. "thin" ≤3 mm). | Arthroscopic group (N = 22):  - Superior one-third: 4 (18%)  - Superior two-thirds: 6 (27%)  - Complete height: 12 (55%)   Open group (N = 13):  - Superior one-third: 3 (23%)  - Superior two-thirds: 6 (46%)  - Complete height: 4 (31%) | Arthroscopic group: Reinsertion from inferior to superior. Horizontal mattress suturing. One anchor for limited superior tears, two or more for larger lesions. Non-absorbable anchors (2 cases) and absorbable anchors (20 cases) (Mitek Worldwide).  Open group: Deltopectoral approach. Horizontal mattress suturing with nonabsorbable anchors (Mitek Worldwide). | Horizontal mattress suturing used in both groups.  Each anchor loaded with two number-2 nonabsorbable sutures.  Suture passage with clever hook (arthroscopic group). | NR | Arthroscopic group (preoperative, N = 22):  - Stage 0 or 1: 19 (86%)  - Stage 2: 3 (14%)  - Stage 3 or 4: 0   Arthroscopic group (postoperative): Progression in 55% (e.g., 0→1, 0→2, 1→2, 1→3, 2→3).  Localized severe fatty infiltration (stages 3–4) in 10 patients (45%) at superior/deep part.   Open group (preoperative, N = 13):  - Stage 0 or 1: 11 (85%)  - Stage 2: 2 (15%)   Open group (postoperative): Progression in 62%; localized severe infiltration in 6 (46%). | Inclusion: Isolated subscapularis tear, arthroscopic or open repair, minimum 2-year clinical follow-up, availability of pre- and postoperative MRI or CTA, no previous shoulder surgery.  Indication: Traumatic or degenerative tear in patients who did not respond to ≥3 months of nonoperative treatment (rest, activity modification, physiotherapy, NSAIDs, corticosteroid injections). Predominant symptoms: pain and weakness.  Exclusions (arthroscopic group): Refused postoperative imaging (1), additional surgery on same shoulder within 2 years (2). | 81.8% (18/22) in arthroscopic group; 92.3% (12/13) in open group | LHBT subluxed or dislocated. Tenodesis in 19 arthroscopic, 13 open; tenotomy in 3 arthroscopic. |
| Oklaz EB., 2024^34^ | - Tear severity: Lafosse classification (Types I–IV).  - LHBT instability: Classified as isolated anterior instability, anterior subluxation, or anterior dislocation (intraoperative dynamic testing).  - Biceps assessment tests: Upper cut test, Yergason test, O'Brien test.  - Subscapularis assessment tests: Lift-off test, belly press test, bear hug test. | By Lafosse classification (N = 20 patients): - Type 2 (complete lesion of superior one-third): 15 (75%) - Type 3 (complete lesion of superior two-thirds): 5 (25%)  (Types 1 and 4 not reported) | Single anchor only (double-loaded bioabsorbable anchor; HEALIX; DePuy Mitek).   Technique: One suture leg passed through subscapularis tendon, other leg of same suture passed through LHBT; repeated with second suture pair. Both repair and tenodesis performed with the same single anchor.   All-arthroscopic, semilateral decubitus position. | Double-loaded anchor (two sutures, each with two legs).  One leg of each suture passed through subscapularis tendon (using suture lasso), other leg of same suture passed through LHBT (using bird beak grasper). Sutures then knotted.   Proximal biceps tendon excised with punch after final knot. | NR | NR | Inclusion: Isolated subscapularis tear (no other rotator cuff pathologies), biceps tenodesis performed for LHBT pathology (instability, partial tear, tenosynovitis, or SLAP lesion), symptoms ≥6 months, failed ≥3 months of physical therapy, minimum 24-month follow-up, adequate accessible data.   Exclusion: Other rotator cuff pathologies, labrum pathology other than SLAP, repair with >1 anchor, biceps tenotomy, acute injury, prior shoulder surgery/fracture, stiff shoulder, chronic/inflammatory arthritis, missing data.   Tenodesis indication: Patient concern for postoperative deformity/residual pain, plus ≥1 of: LHBT dislocation, SLAP lesion, or LHBT subluxation/instability with tenosynovitis/partial tear. | 100% (20/20) (inclusion criteria for tenodesis) | All had tenodesis for LHBT pathology: instability (8 isolated anterior, 10 subluxation, 2 dislocation), SLAP (5), tenosynovitis (13), partial tear (5). |
| Rhee YG., 2017^35^ | - Tear severity: Yoo et al. classification (Types I, IIA, IIB, III, IV, V) based on 3D anatomic footprint and percentage of tendon detachment.  - Fatty infiltration: Fuchs et al. (modified Goutallier for MRI).  - Muscle morphology: CRTD, CATD, CSA measured as described by Scheibel et al. | By Yoo et al. classification (N = 45 patients):  - Type IIA (<50% detachment to first facet, less than one-quarter of entire tendon): 27  - Type IIB (>50% detachment to first facet without complete disruption of lateral band): 12  - Type III (entire first facet exposed, complete thickness tear): 5  - Type IV (first and second facets exposed, more medial retraction): 1  - Type V (complete tear involving muscular portion): 0   Grouped for analysis:  - Group A (Type IIA, <1/4 of tendon): 27  - Group B (Types IIB, III, IV, >1/4 of tendon): 18 | Anchor placed in superior medial portion of first facet. First stitch placed on more medial side for superior stitches; second inferior stitch placed on more lateral side of tissue. Mattress suturing used. Different portal strategies between the two operating surgeons (beach chair vs. semilateral decubitus). | Mattress suturing. One institution: triple-loaded Y-Knot RC; simple stitch with nonsliding knot; alternating half-hitch knots. Other institution: sutures passed with suture hook; both limbs tied with nonsliding knots. | Classified by Yoo et al. system (by percentage of tendon detachment and facets exposed).  Group A: less than one-fourth of entire subscapularis tendon.  Group B: more than one-fourth of entire subscapularis tendon.  No continuous measurement (e.g., cm) provided. | Between groups: Fatty infiltration significantly different between Group A (Type IIA) and Group B (Types IIB/III/IV) (P = .004 for CSA, P = .023 for CRTD, P = .002 for CATD — these reflect structural differences, not Goutallier grade directly; Goutallier grade difference stated but numerical distribution not provided in extracted sections).  Control group comparison: Postoperative CATD same as controls (21.3 vs. 21.0 mm, P = .057); CSA and CRTD not significantly different from controls. | - Clinically symptomatic isolated subscapularis tear.  - Pain for ≥6 months despite conservative treatment (medication).  - Positive in ≥1 of 3 preoperative specific subscapularis function tests.  - Abnormal MRI findings.  - Minimum 24 months follow-up.   Exclusion criteria: Open repairs, tears combined with supraspinatus/infraspinatus, tears combined with other acute/chronic diseases (infection, fractures, osteoarthritis, rheumatoid arthritis, SLE), revision surgery. | Not reported as overall % | LHBT: intact (1), fraying (10), auto loss (11), torn <50% (20), torn >50% (3), subluxated (10), dislocated (1). Tenodesis (13), tenotomy (9), debridement (10). |
| Seppel G., 2017^36^ | - Tear severity: Fox and Romeo classification (Types 1–4).  - Tendon retraction: Patte classification (grades 1–3).  - Fatty infiltration: Goutallier et al. and Fuchs et al. (grades 0–4; >3 excluded).  - Muscle atrophy: CRTD, CATD, vertical diameter, and CSA measured as described by Scheibel et al. and Juul-Kristensen et al./Bartl et al.  - Osteoarthritis: Samilson and Prieto criteria. | By Fox and Romeo classification (N = 17 patients):  - Type 2 (complete tear of upper 25% of tendon): 7 (41.2%)  - Type 3 (complete tear of upper 50% of tendon): 5 (29.4%)  - Type 4 (complete tear of >50% of tendon): 5 (29.4%)   (Type 1 partial tears excluded) | Single-row modified Mason-Allen technique in all cases. Double-loaded suture anchors (5.5mm titanium or bioabsorbable Corkscrew; Arthrex). One anchor for superior tears; ≥2 anchors for larger tears. No routine coracoplasty. No subacromial decompression (supraspinatus not affected). | Modified Mason-Allen technique. Each anchor double-loaded. Specific suture brand not specified (implied from Arthrex anchors). | Classified by Fox and Romeo type (2, 3, 4). Tendon retraction by Patte: grade 1 (8 shoulders, 47.1%), grade 2 (8, 47.1%), grade 3 (1, 5.9%). | Preoperative (Goutallier/Fuchs): Advanced fatty infiltration (grade >3) was an exclusion criterion. 6 patients had cranial SSC atrophy on MRI (no correlation with higher Fox grade reported). | Inclusion: Symptomatic and arthroscopically confirmed isolated full-thickness SSC tears (Fox and Romeo types 2–4).   Exclusion criteria: Partial tears (Fox type 1), concomitant lesions of other rotator cuff tendons, previous surgery on affected shoulder, advanced fatty infiltration (Goutallier >3), advanced osteoarthritis, open repair.   Indication: persistent pain and/or weakness owing to symptomatic SSC tear. | 64.7% (11/17) had tenodesis (10) or tenotomy (1) | 6 patients (35.3%) showed no involvement of LHBT. |
| Yoon JS., 2019^37^ | - Fatty infiltration: Goutallier classification (grades 0–4); study included only grades 3 and 4 ("advanced fatty infiltration"). - Tear severity: Isolated subscapularis full-thickness tear; additionally classified as "reparable" (torn tendon retracted lateral to glenoid on MRI axial view) vs. "irreparable" (retracted medial to glenoid). - Subscapularis strength: Modified belly-press test. | By reparability (N = 52 patients): - Group A (operative, reparable tears): 28 patients - Group B (nonoperative, irreparable tears): 24 patients  By fatty infiltration grade (Goutallier): - Group A: grade 3 (25 patients), grade 4 (3 patients) - Group B: grade 3 (2 patients), grade 4 (22 patients) | Single-row repair ("knots secured in single-row fashion"). Suture anchors inserted through anterior portal; sutures passed in a row via anterolateral working portal using suture passer. Biceps tenotomy performed first in all operative patients; biceps tenotomy or tenodesis performed in all except those with spontaneous rupture. | NR | NR | Inclusion criterion: Goutallier grade 3 or 4 only ("advanced fatty infiltration").  Group A (operative, n=28): grade 3 (25 patients), grade 4 (3 patients) Group B (nonoperative, n=24): grade 3 (2 patients), grade 4 (22 patients)  Postoperative (Group A only): "No progression of fatty infiltration was observed on follow-up MRI scans". | Inclusion (both groups): Isolated subscapularis full-thickness tear with Goutallier grade 3 or 4 advanced fatty infiltration.  Group A (operative): Pain and disability refractory to nonoperative treatment, and tear considered reparable (torn tendon retracted lateral to glenoid on MRI axial view).  Group B (nonoperative): Irreparable tears (torn tendon retracted medial to glenoid).  Exclusion criteria: Lack of minimum 2-year follow-up or ultrasonographic evaluations, severely retracted tear where reattachment of upper tendinous portion not feasible (partial repair of lower portion possible), surgical history on affected shoulder, workers' compensation claims. | Not directly reported (but all operative patients had biceps tenotomy first) | Biceps tendon involvement in all advanced tears; some had spontaneous rupture. |

SSC = Subscapularis, ROM = Range of motion (if used elsewhere in the related section), MRI = Magnetic resonance imaging, LHBT = Long head of the biceps tendon, CTA = Computed tomography arthrography, MRA = Magnetic resonance arthrography

**Additional Table A2. GRADE Evidence Profile for Subscapularis Repair Outcomes**

| **Outcome** | **No. of Studies (Participants)** | **Study Design** | **Risk of Bias** | **Inconsistency** | **Indirectness** | **Imprecision** | **Publication Bias** | **Quality of Evidence (GRADE)** | **Effect Estimate (95% CI)** | **Importance** |
| --- | --- | --- | --- | --- | --- | --- | --- | --- | --- | --- |
| Functional Outcomes |  |  |  |  |  |  |  |  |  |  |
| ASES Score | 7 (249) | Case series, cohort studies | Serious | Very serious | Not serious | Not serious | Not serious | ⊕⊕○○ LOW | MD 48.1 (46.8 to 49.4) | CRITICAL |
| Constant-Murley Score | 14 (variable) | Case series, cohort studies | Serious | Very serious | Not serious | Not serious | Serious | ⊕○○○ VERY LOW | MD 31.4 (27.0 to 35.9) | CRITICAL |
| Simple Shoulder Test | 3 (56) | Case series, cohort studies | Serious | Not serious | Not serious | Serious | Serious | ⊕○○○ VERY LOW | MD 4.9 (4.4 to 5.4) | IMPORTANT |
| Subjective Shoulder Value (%) | 6 (231) | Case series, cohort studies | Serious | Not serious | Not serious | Not serious | Not serious | ⊕⊕○○ LOW | MD 48.2% (46.7 to 49.7) | CRITICAL |
| UCLA Shoulder Score | 6 (154) | Case series, cohort studies | Serious | Not serious | Not serious | Not serious | Not serious | ⊕⊕○○ LOW | MD 15.4 (14.8 to 15.9) | CRITICAL |
| Pain Assessment |  |  |  |  |  |  |  |  |  |  |
| VAS Pain Score | 8 (223) | Case series, cohort studies | Serious | Very serious | Not serious | Not serious | Serious | ⊕○○○ VERY LOW | MD -5.0 (-5.2 to -4.8) | CRITICAL |
| Clinical Tests |  |  |  |  |  |  |  |  |  |  |
| Lift-off Test Resolution | 9 (212 vs 185) | Case series, cohort studies | Serious | Not serious | Not serious | Not serious | Very serious | ⊕○○○ VERY LOW | RR 0.19 (0.14 to 0.27) | IMPORTANT |
| Belly Press Test Resolution | 8 (193) | Case series, cohort studies | Serious | Not serious | Not serious | Not serious | Very serious | ⊕○○○ VERY LOW | RR 0.36 (0.29 to 0.46) | IMPORTANT |
| Bear-hug Test Resolution | 3 (111) | Case series, cohort studies | Serious | Not serious | Not serious | Serious | Not assessed | ⊕○○○ VERY LOW | RR 0.46 (0.35 to 0.61) | IMPORTANT |
| Range of Motion |  |  |  |  |  |  |  |  |  |  |
| External Rotation (degrees) | 14 (variable) | Case series, cohort studies | Serious | Serious | Not serious | Not serious | Not serious | ⊕⊕○○ LOW | MD 5.2° (2.1 to 8.3) | IMPORTANT |
| Internal Rotation - Constant Scale | 6 (141) | Case series, cohort studies | Serious | Serious | Not serious | Not serious | Not serious | ⊕⊕○○ LOW | MD 2.4 (2.0 to 2.7) | IMPORTANT |
| Internal Rotation - Spinal Level | 7 (259) | Case series, cohort studies | Serious | Serious | Not serious | Not serious | Not serious | ⊕⊕○○ LOW | MD -3.5 levels (-3.7 to -3.2) | IMPORTANT |
| Strength Assessment |  |  |  |  |  |  |  |  |  |  |
| Belly-press Strength (MRC Scale) | 7 (164) | Case series, cohort studies | Serious | Very serious | Not serious | Not serious | Serious | ⊕○○○ VERY LOW | MD 1.1 (1.0 to 1.2) | IMPORTANT |
| Lift-off Strength (MRC Scale) | 2 (28) | Case series, cohort studies | Serious | Not serious | Not serious | Very serious | Not assessed | ⊕○○○ VERY LOW | MD 1.8 (1.6 to 2.0) | IMPORTANT |

**Additional Table A3.** Summary of Leave-One-Out Analysis Results

| **Outcome Measure** | **Study Removed** | **Effect Size [95% CI]** | **P-Value** | **I² (%)** | **Clinical Interpretation** |
| --- | --- | --- | --- | --- | --- |
| **ASES Score Change** | None (Overall) | 48.09 [46.76, 49.43] | P < 0.00001 | 95.2% | **Significant improvement maintained** |
|  | Bennett WF., 2003 | 47.96 [46.62, 49.30] | P < 0.00001 | 95.9% |  |
|  | Kamijo H., 2022 | 50.72 [49.27, 52.17] | P < 0.00001 | 87.1% |  |
|  | Kilic AI., 2024 | 49.23 [47.80, 50.66] | P < 0.00001 | 95.2% |  |
|  | Kim SJ., 2018 (CC) | 47.36 [45.88, 48.84] | P < 0.00001 | 95.8% |  |
|  | Kim SJ., 2018 (WC) | 47.25 [45.81, 48.69] | P < 0.00001 | 95.7% |  |
|  | Yoon JS., 2019 (SR) | 47.32 [45.83, 48.81] | P < 0.00001 | 95.8% |  |
|  | Yoon JS., 2019 (DR) | 46.66 [45.17, 48.14] | P < 0.00001 | 95.3% |  |
| **Bear-Hug Test (Risk Ratio)** | None (Overall) | 0.46 [0.35, 0.61] | P < 0.00001 | 0.6% | **Significant reduction in positive tests** |
|  | Kamijo H., 2022 | 0.39 [0.25, 0.60] | P = 0.00002 | 0.8% |  |
|  | Oklaz EB., 2024 | 0.58 [0.44, 0.77] | P = 0.0001 | 0.0% |  |
|  | Rhee YG., 2017 | 0.38 [0.26, 0.55] | P < 0.00001 | 0.8% |  |
| **Belly Press Test (Risk Ratio)** | None (Overall) | 0.36 [0.29, 0.46] | P < 0.00001 | 0.7% | **Significant reduction in positive tests** |
|  | Bartl C., 2011 | 0.38 [0.29, 0.48] | P < 0.00001 | 0.7% |  |
|  | Bennett WF., 2003 | 0.38 [0.30, 0.48] | P < 0.00001 | 0.7% |  |
|  | Heikenfeld R., 2012 | 0.39 [0.31, 0.50] | P < 0.00001 | 0.7% |  |
|  | Kamijo H., 2022 | 0.30 [0.22, 0.41] | P < 0.00001 | 0.7% |  |
|  | Lafosse L., 2007 | 0.38 [0.30, 0.49] | P < 0.00001 | 0.7% |  |
|  | Oklaz EB., 2024 | 0.40 [0.32, 0.51] | P < 0.00001 | 0.7% |  |
|  | Rhee YG., 2017 | 0.26 [0.19, 0.36] | P < 0.00001 | 0.6% |  |
|  | Seppel G., 2017 | 0.38 [0.30, 0.49] | P < 0.00001 | 0.7% |  |
| **Constant-Murley Score** | None (Overall) | 31.40 [26.95, 35.85] | P = 0.000 | 92.7% | **Significant functional improvement** |
|  | Bennett WF., 2003 | 31.43 [26.82, 36.04] | P = 0.000 | 93.3% |  |
|  | Grueninger P., 2014 | 30.25 [25.78, 34.72] | P = 0.000 | 92.2% |  |
|  | Bartl C., 2011 | 31.31 [26.41, 36.20] | P = 0.000 | 93.2% |  |
|  | Hasler A., 2019 | 32.50 [28.16, 36.85] | P = 0.000 | 92.1% |  |
|  | All other studies | 30.54-32.43 | P = 0.000 | 90.5-93.3% |  |
| **External Rotation Score** | None (Overall) | 5.16 [2.05, 8.26] | P = 0.001 | 80.0% | **Modest but significant improvement** |
|  | Godinho GG., 2015 | 6.12 [3.25, 8.98] | P = 0.000 | 75.3% |  |
|  | Kamijo H., 2022 | 5.42 [2.10, 8.74] | P = 0.001 | 81.0% |  |
|  | All other studies | 4.05-5.47 | P = 0.001-0.005 | 68.7-81.5% |  |
| **Internal Rotation (Spinal Level)** | None (Overall) | -3.45 [-3.74, -3.16] | P < 0.00001 | 76.4% | **Significant improvement** |
|  | Godinho GG., 2015 | -3.38 [-3.68, -3.08] | P < 0.00001 | 75.5% |  |
|  | Kamijo H., 2022 | -3.64 [-3.95, -3.33] | P < 0.00001 | 62.3% |  |
|  | All other studies | -3.32 to -3.56 | P < 0.00001 | 77.5-78.7% |  |
| **Internal Rotation (Constant-Murley)** | None (Overall) | 2.38 [2.03, 2.74] | P < 0.00001 | 80.9% | **Significant improvement** |
|  | Bartl C., 2024 | 2.49 [2.09, 2.89] | P < 0.00001 | 84.0% |  |
|  | Galasso O., 2024 | 1.94 [1.54, 2.35] | P < 0.00001 | 17.4% |  |
|  | All other studies | 2.32-2.61 | P < 0.00001 | 80.2-84.2% |  |
| **Lift-off Test (Risk Ratio)** | None (Overall) | 0.19 [0.14, 0.27] | P < 0.00001 | 0.4% | **Significant reduction in positive tests** |
|  | All individual studies | 0.14-0.21 | P < 0.00001 | 0.0-0.5% |  |
| **SST Score** | None (Overall) | 4.93 [4.41, 5.45] | P < 0.00001 | 0.0% | **Significant strength improvement** |
|  | Bartl C., 2024 | 4.75 [4.01, 5.49] | P < 0.00001 | 0.0% |  |
|  | Bartl C., 2011 | 4.94 [4.29, 5.59] | P < 0.00001 | 0.0% |  |
|  | Seppel G., 2017 | 5.02 [4.46, 5.57] | P < 0.00001 | 0.0% |  |
| **SSV (%)** | None (Overall) | 48.20 [46.65, 49.75] | P < 0.00001 | 38.9% | **Significant functional improvement** |
|  | All individual studies | 47.89-49.02 | P < 0.00001 | 0.0-50.8% |  |
| **UCLA Score** | None (Overall) | 15.37 [14.80, 15.94] | P < 0.00001 | 0.0% | **Significant functional improvement** |
|  | All individual studies | 15.16-15.62 | P < 0.00001 | 0.0% |  |
| **VAS Pain Score** | None (Overall) | -5.03 [-5.22, -4.83] | P < 0.00001 | 91.3% | **Significant pain reduction** |
|  | All individual studies | -4.82 to -5.28 | P < 0.00001 | 85.9-92.5% |  |
| **Belly-press Strength (MRC)** | None (Overall) | 1.11 [1.03, 1.19] | P < 0.00001 | 96.5% | **Significant strength improvement** |
|  | All individual studies | 0.99-1.18 | P < 0.00001 | 94.8-97.0% |  |

ASES, American Shoulder and Elbow Surgeons Score; CC, concomitant coracoplasty; DR, Double Row; F: female; M: male; SD, standard deviation; SR, Single Row; SSV: Subjective Shoulder Value; WC, without coracoplasty.

**Additional Table A4. Functional Outcome Scores Assessment**

| **Study (first author, year)** | **CMS**  **(n)** **mean ± SD** | | **UCLA**  **(n)** **mean ± SD** | | **ASES**  **(n)** **mean ± SD** | | **SST**  **(n)** **mean ± SD** | | **SSV (%)**  **(n)** **mean ± SD** | | **VAS**  **(n)** **mean ± SD** | |
| --- | --- | --- | --- | --- | --- | --- | --- | --- | --- | --- | --- | --- |
|  | Pre-op | Post-op | Pre-op | Post-op | Pre-op | Post-op | Pre-op | Post-op | Pre-op | Post-op | Pre-op | Post-op |
| Bartl C., 2024 | (18) 54.0 ± 7.0 | (18) 86.0 ± 6.0 | N/R | N/R | N/R | N/R | (18) 5.8 ± 1.7 | (18) 10.9 ± 1.4 | N/R | N/R | N/R | N/R |
| Bartl C., 2011 | (21) 50.3 ± 7.5 | (21) 82.4 ± 8.6 | N/R | N/R | N/R | N/R | (21) 6.3 ± 2.3 | (21) 11.2 ± 1.4 | N/R | N/R | N/R | N/R |
| Bennett WF., 2003 | (8) 43.3 ± 14.1 | (8) 74.2 ± 16.8 | N/R | N/R | (8) 16.1 ± 16.2 | (8) 74.4 ± 17.6 | N/R | N/R | N/R | N/R | (8) 9.0 ± 1.6 | (8) 2.0 ± 1.6 |
| Galasso O., 2024 | (45) 45.3 ± 7.5 | (45) 86.7 ± 8.6 | N/R | N/R | N/R | N/R | N/R | N/R | N/R | N/R | N/R | N/R |
| Godinho GG., 2015 | N/R | N/R | N/R | N/R | N/R | N/R | N/R | N/R | N/R | N/R | N/R | N/R |
| Grueninger P., 2014 | (11) 43.5 ± 7.5 | (11) 89.3 ± 7.8 | N/R | N/R | N/R | N/R | N/R | N/R | N/R | N/R | N/R | N/R |
| Hasler A., 2019 | (36) 60.6 ± 14.1 | (36) 81.0 ± 8.5 | N/R | N/R | N/R | N/R | N/R | N/R | (36) 40.0 ± 25.0 | (36) 91.0 ± 13.0 | N/R | N/R |
| Heikenfeld R., 2012 | (20) 41.3 ± 7.5 (19) 41.3 ± 7.5 | (20) 80.2 ± 7.8 (19) 81.0 ± 8.3 | (19) 16.5 ± 5.5 | (19) 32.5 ± 3.8 | N/R | N/R | N/R | N/R | N/R | N/R | N/R | N/R |
| Kamijo H., 2022 | N/R | N/R | N/R | N/R | (46) 56.0 ± 12.0 | (46) 89.0 ± 12.0 | N/R | N/R | N/R | N/R | N/R | N/R |
| Kilic AI., 2024 | N/R | N/R | N/R | N/R | (77) 41.5 ± 16.2 | (77) 81.6 ± 17.6 | N/R | N/R | (77) 38.2 ± 19.9 | (77) 80.5 ± 19.9 | (77) 5.8 ± 1.9 | (77) 1.9 ± 2.1 |
| Kim SJ., 2018 (CC) | N/R | N/R | (35) 16.8 ± 3.3 | (35) 31.3 ± 4.0 | (35) 39.1 ± 8.7 | (35) 90.4 ± 9.9 | N/R | N/R | (35) 40.9 ± 9.5 | (35) 89.4 ± 8.9 | (35) 6.0 ± 1.5 | (35) 1.3 ± 1.3 |
| Kim SJ., 2018 (WC) | N/R | N/R | (27) 16.4 ± 2.9 | (27) 31.5 ± 4.6 | (27) 37.7 ± 6.4 | (27) 90.8 ± 10.7 | N/R | N/R | (27) 41.9 ± 8.3 | (27) 90.7 ± 10.7 | (27) 6.1 ± 1.3 | (27) 1.2 ± 1.3 |
| Lafosse L., 2007 | (17) 52.0 ± 14.1 | (17) 84.9 ± 16.8 | (17) 16.2 ± 5.5 | (17) 32.1 ± 4.6 | N/R | N/R | N/R | N/R | N/R | N/R | N/R | N/R |
| Nove-Josserand L., 2012 | (22) 66.4 ± 14.1 | (22) 85.2 ± 16.8 | N/R | N/R | N/R | N/R | N/R | N/R | N/R | N/R | N/R | N/R |
| Oklaz EB., 2024 (M) | (10) 57.1 ± 11.9 | (10) 87.4 ± 5.5 | N/R | N/R | N/R | N/R | N/R | N/R | N/R | N/R | (10) 7.6 ± 1.1 | (10) 0.8 ± 0.7 |
| Oklaz EB., 2024 (F) | (10) 57.6 ± 11.6 | (10) 86.0 ± 3.4 | N/R | N/R | N/R | N/R | N/R | N/R | N/R | N/R | (10) 7.5 ± 0.9 | (10) 1.3 ± 1.6 |
| Rhee YG., 2017 | N/R | N/R | N/R | N/R | N/R | N/R | N/R | N/R | N/R | N/R | N/R | N/R |
| Seppel G., 2017 | (17) 47.8 ± 21.3 | (17) 74.2 ± 21.3 | N/R | N/R | N/R | N/R | (17) 6.2 ± 3.4 | (17) 10.5 ± 2.6 | N/R | N/R | N/R | N/R |
| Yoon JS., 2019 (SR) | N/R | N/R | (31) 16.5 ± 3.3 | (31) 31.9 ± 3.5 | (31) 39.7 ± 8.8 | (31) 91.0 ± 8.4 | N/R | N/R | (31) 41.9 ± 10.5 | (31) 91.3 ± 8.6 | (31) 6.0 ± 1.6 | (31) 1.2 ± 1.1 |
| Yoon JS., 2019 (DR) | N/R | N/R | (25) 16.2 ± 2.7 | (25) 32.1 ± 2.8 | (25) 37.2 ± 6.4 | (25) 91.4 ± 8.7 | N/R | N/R | (25) 42.8 ± 9.4 | (25) 91.8 ± 8.8 | (25) 6.2 ± 1.3 | (25) 1.1 ± 1.0 |

ASES = American Shoulder and Elbow Surgeons Score; CMS = Constant-Murley Score; N/R = Not Reported; SST = Simple Shoulder Test; SSV = Subjective Shoulder Value; UCLA = University of California Los Angeles Shoulder Score; VAS = Visual Analog Scale

**Additional Table A5.** Range of Motion and Strength Assessment

| **Study (first author, year)** | **Internal Rotation** | | |  | **Internal Rotation Strength (MRC Scale 0-5)** | | | | | | | | |  |
| --- | --- | --- | --- | --- | --- | --- | --- | --- | --- | --- | --- | --- | --- | --- |
|  | Assessment Method | Pre-op (n) mean ± SD | | Post-op (n) mean ± SD | Scale | | Lift-off Pre-op (n) mean ± SD | | Lift-off Post-op (n) mean ± SD | | Belly-press Pre-op (n) mean ± SD | | Belly-press Post-op (n) mean ± SD | |
| Bartl C., 2024 | Constant-Murley IR | (18) 5.6 ± 1.8 | | (18) 7.6 ± 1.5 | 0-10 points | | N/R | | N/R | | (18) 2.3 ± 0.5 | | (18) 4.5 ± 0.6 | |
| Bartl C., 2011 | N/R | N/R | | N/R | N/R | | N/R | | N/R | | N/R | | N/R | |
| Bennett WF., 2003 | N/R | N/R | | N/R | N/R | | N/R | | N/R | | N/R | | N/R | |
| Galasso O., 2024 | Constant-Murley IR | (45) 3.0 ± 2.0 | | (45) 7.0 ± 3.0 | 0-10 points | | N/R | | N/R | | N/R | | N/R | |
| Godinho GG., 2015 | Spinal Level | (18) 12.0 ± 3.0 | | (18) 7.0 ± 3.0 | 10-20 points | | N/R | | N/R | | N/R | | N/R | |
| Grueninger P., 2014 | N/R | N/R | | N/R | N/R | | (11) 2.9 ± 0.4 | | (11) 4.8 ± 0.6 | | (11) 2.9 ± 0.3 | | (11) 4.8 ± 0.6 | |
| Hasler A., 2019 | N/R | N/R | | N/R | N/R | | N/R | | N/R | | N/R | | N/R | |
| Heikenfeld R., 2012 | N/R | N/R | | N/R | N/R | | N/R | | N/R | | N/R | | N/R | |
| Kamijo H., 2022 | Spinal Level | (46) 14.0 ± 3.0 | | (46) 12.0 ± 3.0 | 10-20 points | | N/R | | N/R | | N/R | | N/R | |
| Kilic AI., 2024 | Spinal Level | (77) 16.0 ± 3.0 | | (77) 12.0 ± 3.0 | 10-20 points | | N/R | | N/R | | N/R | | N/R | |
| Kim SJ., 2018 (CC) | Spinal Level | (35) 14.0 ± 2.0 | | (35) 11.0 ± 2.0 | 10-20 points | | N/R | | N/R | | (35) 3.8 ± 0.4 | | (35) 4.6 ± 0.6 | |
| Kim SJ., 2018 (WC) | Spinal Level | (27) 15.0 ± 2.0 | | (27) 11.0 ± 2.0 | 10-20 points | | N/R | | N/R | | (27) 3.8 ± 0.4 | | (27) 4.6 ± 0.6 | |
| Lafosse L., 2007 | Constant-Murley IR | (22) 6.1 ± 2.1 | | (22) 7.6 ± 1.6 | 0-10 points | | (17) 2.4 ± 0.4 | | (17) 4.1 ± 0.6 | | (17) 2.5 ± 0.5 | | (17) 4.4 ± 0.6 | |
| Nove-Josserand L., 2012 | N/R | N/R | | N/R | N/R | | N/R | | N/R | | N/R | | N/R | |
| Oklaz EB., 2024 (M) | Constant-Murley IR | (10) 5.2 ± 2.1 | (10) 8.2 ± 1.4 | 0-10 points | | N/R | | N/R | | N/R | | N/R | |  |
| Oklaz EB., 2024 (F) | Constant-Murley IR | (10) 6.0 ± 2.1 | (10) 7.6 ± 1.2 | 0-10 points | | N/R | | N/R | | N/R | | N/R | |  |
| Rhee YG., 2017 | N/R | N/R | N/R | N/R | | N/R | | N/R | | N/R | | N/R | |  |
| Seppel G., 2017 | N/R | N/R | N/R | N/R | | N/R | | N/R | | N/R | | N/R | |  |
| Yoon JS., 2019 (SR) | Spinal Level | (31) 14.0 ± 2.0 | (31) 11.0 ± 2.0 | 10-20 points | | N/R | | N/R | | (31) 3.8 ± 0.4 | | (31) 4.7 ± 0.5 | |  |
| Yoon JS., 2019 (DR) | Spinal Level | (25) 15.0 ± 2.0 | (25) 11.0 ± 2.0 | 10-20 points | | N/R | | N/R | | (25) 3.9 ± 0.5 | | (25) 4.6 ± 0.5 | |  |

IR = Internal Rotation; MRC = Medical Research Council; N/R = Not Reported

**Additional Figures**

**Additional Figure A1**. Mean change in SSV (A) and VAS (B) from preoperative to postoperative. CC, concomitant coracoplasty; DR, Double Row; F: female; M: male; SD, standard deviation; SR, Single Row; WC, without coracoplasty.

**Additional Figure A2**. Funnel Plot showing no publication bias for a) CMS score, b) ASES score, c) SST score, d) SSV score and e) UCLA score.

**Additional Figure A3**. Funnel plots of a) VAS score, b) Lift-off score, c) Belly Press and d) Belly-press Strength score, showing publication bias

**Additional Figure A4**. Mean change in Lift-off (A), Belly Press (B) and Bear Hug (C) from preoperative to postoperative.

**Additional Figure A5**. Funnel Splots of a) Bear-Hug test, b) Internal Rotation (Constant-Murley Scale) and c) Internal Rotation (Spinal Level Scales).

**Additional Figure A6**. Mean change in Internal Rotation (Constant-Murley Scale, 0-10 points) from preoperative to postoperative. SD, standard deviation; F: female; M: male.

**Additional Figure A7**. Mean change in Internal Rotation (Spinal Level Scale, 10-20 points) from preoperative to postoperative. CC, concomitant coracoplasty; DR, Double Row; F: female; M: male; SD, standard deviation; SR, Single Row; WC, without coracoplasty.

**Additional Figure A8**. Mean change in External Rotation Score (ER) from preoperative to postoperative. CC, concomitant coracoplasty; DR, Double Row; F: female; M: male; SD, standard deviation; SR, Single Row; WC, without coracoplasty.

**Additional Figure A9**. Mean change in Internal Rotation Strength (Belly-press Strength MRC Scale Analysis) from preoperative to postoperative. CC, concomitant coracoplasty; DR, Double Row; SD, standard deviation; SR, Single Row; WC, without coracoplasty.

**Additional Figure A10**. Mean change in Internal Rotation Strength (Lift-off Strength MRC Scale Analysis) from preoperative to postoperative.
